# Depletion of a *Toxoplasma* porin leads to defects in mitochondrial morphology and contacts with the endoplasmic reticulum

**DOI:** 10.1242/jcs.255299

**Published:** 2021-10-20

**Authors:** Natalia Mallo, Jana Ovciarikova, Erica S. Martins-Duarte, Stephan C. Baehr, Marco Biddau, Mary-Louise Wilde, Alessandro D. Uboldi, Leandro Lemgruber, Christopher J. Tonkin, Jeremy G. Wideman, Clare R. Harding, Lilach Sheiner

**Affiliations:** 1 Wellcome Centre for Integrative Parasitology, University of Glasgow, Glasgow G12 8TA, UK; 2 Departamento de Parasitologia, Instituto de Ciências Biológicas, Universidade Federal de Minas Gerais, Belo Horizonte 486 31270-901, Brazil; 3 Biodesign Center for Mechanisms of Evolution, School of Life Sciences, Arizona State University, Tempe, AZ 85287, USA; 4 The Walter and Eliza Hall Institute of Medical Research, Melbourne, VIC 3086, Australia; 5 Department of Medical Biology, The University of Melbourne, Melbourne, VIC 3010, Australia; 6 Glasgow Imaging Facility, University of Glasgow, Glasgow G12 8TA, UK

**Keywords:** VDAC, Porin, Mitochondria, Mitochondrion, ER, Ca^2+^, Toxoplasma, Motility, Membrane contact sites

## Abstract

The voltage-dependent anion channel (VDAC) is a ubiquitous channel in the outer membrane of the mitochondrion with multiple roles in protein, metabolite and small molecule transport. In mammalian cells, VDAC protein, as part of a larger complex including the inositol triphosphate receptor, has been shown to have a role in mediating contacts between the mitochondria and endoplasmic reticulum (ER). We identify VDAC of the pathogenic apicomplexan *Toxoplasma gondii* and demonstrate its importance for parasite growth. We show that VDAC is involved in protein import and metabolite transfer to mitochondria. Further, depletion of VDAC resulted in significant morphological changes in the mitochondrion and ER, suggesting a role in mediating contacts between these organelles in *T. gondii*.

This article has an associated First Person interview with the first author of the paper.

## Abstract (precis)

**Summary:** Depletion of the *Toxoplasma* voltage-dependent anion channel highlights the importance of endoplasmic reticulum–mitochondria membrane contact sites in maintaining organelle morphology.

## INTRODUCTION

*Toxoplasma gondii* is the causative agent of toxoplasmosis and a member of the parasitic apicomplexan family, which includes *Plasmodium*, the causative agent of malaria, and *Cryptosporidium*, which causes the diarrheal disease cryptosporidiosis. As a divergent eukaryote, *T. gondii* has a single, lasso-shaped mitochondrion, which is required for respiration and metabolism. To enable this, multiple small molecules, metabolites and proteins must cross the two membranes of the mitochondrion. In many eukaryotes, from protists to mammals, transport across the outer mitochondrial membrane is mediated by a conserved, highly abundant porin named the voltage-dependent anion channel (VDAC) (Hodge and Colombini, 1997; Homblé et al., 2012; Wideman et al., 2013). VDAC mediates the passage of ions, nucleotides and metabolites across the outer membrane, and has been implicated in protein and tRNA import into the mitochondria (Camara et al., 2017; Ellenrieder et al., 2019; Hodge and Colombini, 1997; Salinas et al., 2006). Beyond mediating transfer across the outer membrane, VDAC has also been identified as a component of membrane contact sites between the mitochondria and endoplasmic reticulum (ER). VDAC clusters in domains of ER–mitochondria contacts (Rapizzi et al., 2002; Shoshan-Barmatz et al., 2004), where it interacts directly with the ER-resident inositol trisphosphate receptor (IP3R) via the chaperone Grp75 (Honrath et al., 2017; Szabadkai et al., 2006). This close apposition of membranes allows direct transfer of Ca^2+^ between the ER and the mitochondria, where the close association of the mitochondrial inner membrane Ca^2+^ uniporter (MCU) and associated protein MICU on the inner membrane of the mitochondria allows uptake of Ca^2+^ into the organelle (De Stefani et al., 2016; Liao et al., 2015). This contact has been shown to have important roles in Ca^2+^ homeostasis in survival and proliferation in mammalian cells (De Stefani et al., 2016; Rieusset et al., 2016; Rizzuto et al., 2009).

In *T. gondii*, the ER is considered the major Ca^2+^ store, and key aspects of the parasite's life cycle, such as host-cell invasion and gliding motility, are initiated and regulated by Ca^2+^ release, largely thought to be from the ER (Borges-Pereira et al., 2015; Lourido and Moreno, 2015; Lovett and Sibley, 2003; Wetzel et al., 2004). However, there is evidence that other organelles, including the mitochondrion and acidocalcisomes, also play a role in Ca^2+^ storage in Apicomplexa (Miranda et al., 2010; Nagamune et al., 2007; Rohloff et al., 2011), and the relative importance of these potential stores in modulating invasion and motility remains unknown.

Establishment and maintenance of the *T. gondii* mitochondrial morphology involves dynamic contacts with other organelles. The abundance of contacts between the mitochondrion and the parasite pellicles [or inner membrane complex (IMC)] correlates with mitochondrial positioning at the periphery (Ovciarikova et al., 2017). Recently, the mitochondrial membrane protein LMF1 was proposed to mediate mitochondrion–IMC contacts, supported by its demonstrated essentiality for mitochondrial peripheral distribution (Jacobs et al., 2020). LMF1 interacts with the fission protein TgFis1, which is also essential for mitochondrial morphology and biogenesis (Jacobs et al., 2020; Melatti et al., 2019), suggesting an involvement of both proteins in these mitochondrial–IMC contacts, while the IMC counterparts have yet to be reported. In contrast, there has been no progress in identifying players in contacts between the mitochondrion and other organelles, including the ER in *T. gondii*. Given its conserved roles in diverse eukaryotes, it is possible that VDAC plays a role in mediating membrane contacts in *T. gondii*.

Here, we identified and characterised VDAC of *T. gondii*. We found that VDAC is important for fitness, and its depletion results in changes in mitochondrial and ER morphology in concert with reduced ER–mitochondrion contacts, suggesting, for the first time, the existence of mitochondrial–ER contact sites in this parasite. Interestingly, this was accompanied by only a mild change in cytosolic Ca^2+^ in response to a phosphodiesterase inhibitor, while processes that depend on large changes in cytosolic Ca^2+^, such as parasite motility and invasion, remained unchanged, pointing to a divergence between parasites and mammalian cells, in which VDAC has a more central role in Ca^2+^ homeostasis.

## RESULTS

### *T. gondii* VDAC is required for parasite growth

**Fig. 1.**
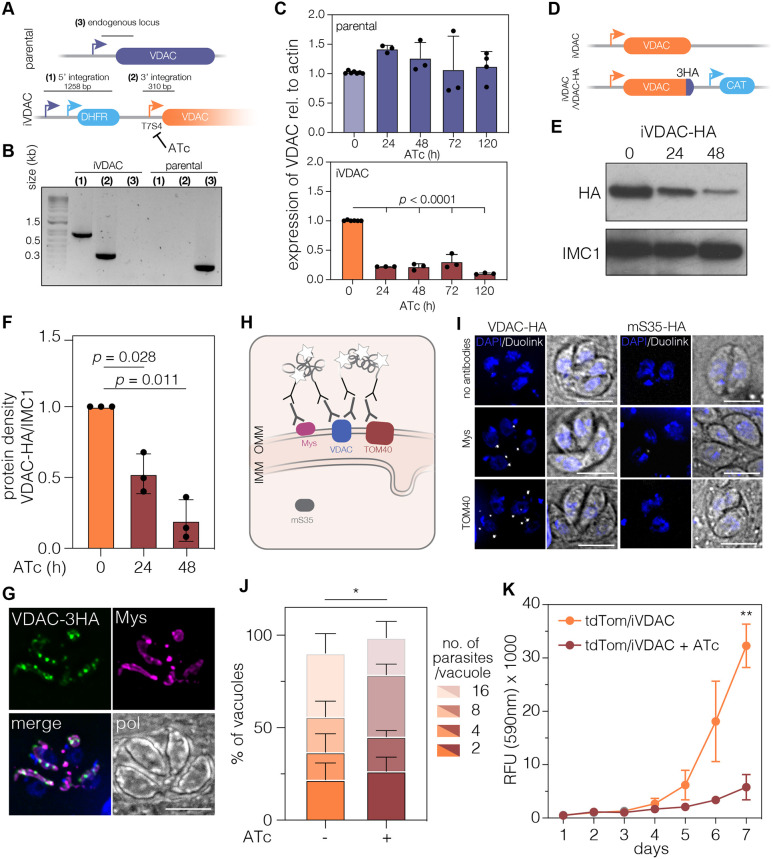
**Downregulation of VDAC leads to decrease in parasite replication and growth.** (A) Schematic representation of the promoter replacement strategy used to create the inducible VDAC (iVDAC) parasite line. The expected sizes of the integration PCRs are given. (B) PCRs to confirm integration of the regulatable promoter and the DHFR selectable marker cassette and the lack of the unmodified gene. (C) Expression of *VDAC* relative to *actin* measured by RT-qPCR over anhydrotetracycline (ATc) treatment duration. Bars represent mean+s.d., statistical analysis performed with one-way ANOVA with Tukey correction, *n*>3 independent experiments. (D) Schematic representation of tagging strategy to endogenously tag VDAC in the iVDAC line. (E) Western blot of iVDAC parasites treated for 24 h and 48 h with ATc. Blots probed with anti-HA to visualise VDAC-HA and anti-IMC as a loading control. Representative of three independent experiments. (F) Quantification of protein quantity by densitometry. Each point represents a replicate, normalised to IMC1, bars at mean±s.d., *n*=3. (G) Endogenously tagged VDAC colocalises with the mitochondrial outer membrane protein Mys (pol, polarised light). Scale bar: 5 µm. (H) Schematic showing mechanism of DuoLink assay and expected localisation of proteins tested. IMM, inner mitochondrial membrane; OMM, outer mitochondrial membrane. (I) DuoLink assay micrographs showing that signal (white) is formed when antibodies against the outer membrane proteins Mys and TOM40 are used together with anti-HA, recognising VDAC. However, no signal is detected using the HA-tagged matrix protein mS35 and Mys/TOM40. Scale bars: 5 µm. (J) Replication assay in which the number of parasites per vacuole was quantified at 48 h post-ATc addition. There were significantly (*P<0.05, unpaired Student's *t*-test, *n*=4) fewer vacuoles containing eight and 16 parasites upon treatment with ATc than the control. (K) Fluorescence of iVDAC parasites stably expressing tdTomato±ATc over time. Results are mean fluorescence (arbitrary units)±s.d., **P=0.005, unpaired Student's *t*-test corrected for multiple tests using Holm-Sidak, *n*=3.

The gene encoding *T. gondii* VDAC (TGME49_263300, XP_002365430.1) was previously identified (Wideman et al., 2013). Using HHPRED, TGME49_263300 had high (E-value=2.2 e^−39^) structural homology to mammalian VDAC, including a conserved glutamate at position 16, which has previously been shown to have a role in voltage conductance (Fig. S1A) (Shuvo et al., 2016). Gene ontology suggested anion transport and voltage-gated channel activity, and data from the recent *T. gondii* protein atlas predicted mitochondrial localisation, together supporting VDAC orthology (Barylyuk et al., 2020; Gajria et al., 2008). We thus named TGME49_263300 VDAC. VDAC is conserved between Apicomplexa, and although sequence identity between orthologues can drop below 30% (Fig. S1B), VDAC is nearly ubiquitously conserved across all eukaryotes (Wideman et al., 2013). Results from genome-wide CRISPR screens suggested that VDAC was important for fitness in *T. gondii*, with a fitness score of −3.57 (Sidik et al., 2016). Based on this, and to examine the role of VDAC in the lifecycle in more detail, we constructed a conditional knockdown line (named iVDAC for ‘inducible VDAC’) by replacing the VDAC promoter with a T7S4 promoter, which is repressed upon addition of anhydrotetracycline (ATc) (Lacombe et al., 2019; Sheiner et al., 2011) (Fig. 1A). Integration of the regulatable promotor was confirmed by PCR (Fig. 1B). Downregulation of the corresponding mRNA upon ATc addition was assessed by quantitative RT-PCR (RT-qPCR), demonstrating an 80% reduction in mRNA levels at 24 h post-treatment, which remains for at least 5 days (Fig. 1C). To visualise VDAC within the parasite, we endogenously tagged VDAC at the C-termini with 3HA (Fig. 1D). Using this tagged line (called iVDAC-HA), we assessed protein depletion after ATc treatment and saw a significant decrease in VDAC at 48 h (Fig. 1E). We quantified this depletion and found an ∼80% decrease in VDAC-HA at 48 h post-ATc treatment (Fig. 1F).

Using endogenously tagged VDAC, we saw VDAC localised to the mitochondrion in *T. gondii*, as demonstrated by colocalisation with the mitochondrion outer membrane marker TgMys (Ovciarikova et al., 2017) (Fig. 1G). Interestingly, tagged-VDAC was not distributed smoothly along the mitochondrion but instead was concentrated in specific patches. This non-uniform localisation has been observed in mammalian cells in which VDAC forms clusters at ER–mitochondrial contact sites (Rapizzi et al., 2002). Interestingly, endogenous tagging of VDAC led to altered mitochondrial morphology. To confirm VDAC localisation, we also cloned the predicted mRNA sequence into a *T. gondii* expression vector with a strong promotor and N-terminal Myc epitope tag (Harding et al., 2016). Parasites transiently expressing this exogenous copy were imaged, and Myc-VDAC demonstrated the same localisation (Fig. S2A), confirming that the tagging location did not affect the protein's localisation. To assess whether VDAC was localised in the outer mitochondrial membranes, we made use of the DuoLink proximity ligation amplification (PLA) system, which has previously been used successfully in *T. gondii* (Long et al., 2017). This allows for amplification and detection of a signal only when the proteins, recognised by antibodies, are in close proximity (Fig. 1H). DuoLink has previously been used successfully to examine the orientation of proteins in the membrane (Ivanusic et al., 2014). To determine whether VDAC was found on the outer mitochondrial membrane, we used endogenously tagged VDAC and antibodies against two proteins known to be in the outer mitochondrial membrane, Mys (Ovciarikova et al., 2017) and TOM40 (van Dooren et al., 2016). We saw specific amplification signals for both (Fig. 1I; Fig. S2B). In contrast, applying DuoLink PLA with the endogenously tagged mitochondrial ribosomal protein mS35-HA, expected to localise to the mitochondrial matrix (Lacombe et al., 2019), and Mys and TOM40 produced no signal above background (Fig. 1I). Together, these observations suggest that, as in other organisms, VDAC is localised in the outer mitochondrial membrane.

VDAC is predicted to be essential in *T. gondii*. To assess the replication of these parasites, we quantified the number of parasites/vacuole at 48 h post-ATc addition. We saw a significant decrease in the numbers of vacuoles with eight and 16 parasites, indicative of a replication defect (Fig. 1J). To confirm this, we performed a fluorescent growth assay, quantifying parasite fluorescence every day for 7 days. Growth of fluorescent iVDAC parasites was significantly inhibited by 7 days post-ATc addition (Fig. 1K), demonstrating that VDAC is important for parasite replication.

### VDAC depletion does not affect resting cytosolic Ca^2+^, or Ca^2+^-mediated stages of the lytic lifecycle

**Fig. 2.**
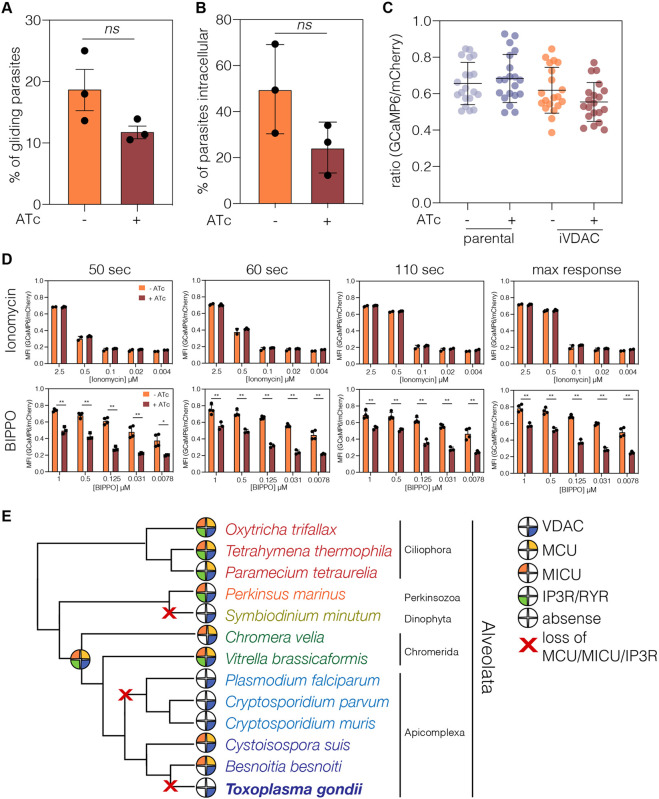
**VDAC depletion does not affects gliding motility and has no effect on resting cytosolic Ca^2+^.** (A) The percentage of iVDAC parasites with trails was quantified. Bars represent mean±s.d., unpaired Student's *t*-test, *n*=3. ns, no significant difference. (B) Percentage of iVDAC parasites internalised after 20 min, unpaired Student's *t*-test, *n*=3. (C) Parental and iVDAC parasites expressing mCherry and GCaMP were analysed by flow cytometry, and the GCaMP/mCherry ratio was calculated in resting parasites. No significant difference could be seen in the ratio upon depletion of VDAC. Bars represent mean±s.d., *n*=4. (D) GCaMP/mCherry ratio was calculated after stimulation with various concentrations of ionomycin or BIPPO for the indicated time. There was no change in GCaMP/mCherry ratio upon stimulation with ionomycin; however, there was a significant decrease in the GCaMP/mCherry ratio in ATc-treated parasites upon treatment with BIPPO. Results from three independent experiments; *P<0.05, **P<0.005, values from multiple *t*-tests with Benjamini correction. (E) Putative orthologues of VDAC, MCU, MICU and IP3R were identified by the reciprocal BLAST method. Filled pies indicate presence of a particular orthologue. Historical losses of all of MCU, MICU, and IP3R are indicated with red ‘X’s. Although VDAC was not identified in *Symbiodinium minutum* (likely due to the incomplete database), it was identified in a closely related species, *Symbiodinium microadriaticum*.

There is evidence that VDAC is involved in Ca^2+^ mobilisation from the mitochondria in other organisms (Honrath et al., 2017; Min et al., 2012; Rieusset et al., 2016; Szabadkai et al., 2006). Both gliding motility and invasion are regulated by transient changes in cytosolic Ca^2+^ levels. To determine whether VDAC depletion affected these processes, we quantified the percentage of gliding (Fig. 2A) and invaded (Fig. 2B) parasites after 72 h of ATc treatment. We saw no significant change in either gliding or invasion, providing further support that the lack of growth in Fig. 1J and K is driven by a replication defect.

We then analysed cytosolic [Ca^2+^] levels using the genetically encoded Ca^2+^ sensor GCaMP6, normalised to mCherry fluorescence, as previously described (Stewart et al., 2017). If VDAC is responsible for mediating [Ca^2+^] mobilisation, it is possible that this would be reflected in changes in the cytosolic [Ca^2+^]. However, we saw no change in GCaMP6 fluorescence upon depletion of VDAC (Fig. 2C). To investigate the response to Ca^2+^ store release under VDAC depletion, GCaMP6-expressing parasites were stimulated with increasing concentrations of either the Ca^2+^ ionophore ionomycin or the specific phosphodiesterase inhibitor 5-benzyl-3-isopropyl-1H-pyrazolo[4,3-d]pyrimidin-7(6H)-one (BIPPO), which increases levels of cGMP, leading to activation of PKG-dependent signalling and a downstream rise in cytosolic [Ca^2+^] (Stewart et al., 2017). In both cases, treatment with the drug resulted in a concentration-dependent increase in GCaMP6 signal, demonstrating effective release of Ca^2+^ stores (Fig. 2D). Only a mild decrease in GCaMP6 signal was seen upon depletion of VDAC following BIPPO treatment, indicating that VDAC likely does not have a central role in stimulated Ca^2+^ release under these conditions, although given the residual VDAC still expressed at this time point (Fig. 1E), we cannot categorically exclude a role for VDAC.

In animals, VDAC mediates Ca^2+^ transfer from the ER to the mitochondria through association with the ER-localised IP3R via Grp75 in the cytosol (Min et al., 2012; Szabadkai et al., 2006). MCU and associated protein MICU interact with VDAC and are also required for Ca^2+^ transfer across the mitochondrial membranes (De Stefani et al., 2016; Rizzuto et al., 2009). Although VDAC and Grp75 are ubiquitous across eukaryotes (Wideman et al., 2013), apicomplexans and multiple other lineages are reported to lack MCU, MICU and IP3Rs [or the paralogous ryanodine receptors (RYRs)] (Bick et al., 2012). On the basis of a number of new genomes becoming available, we re-investigated the presence of MCU, MICU and IP3R/RYR3 in alveolates (Fig. 2E) and wider eukaryotes (Fig. S3). We found no evidence for MCU, MICU or IP3R/RYR3 in *T. gondii*, *Plasmodium falciparum* or *Cryptosporidium* spp. (Fig. 2E). However, we identified orthologues of MCU and MICU in *Besnoitia besnoiti* and *Cystoisospora suis*, parasites closely related to *T. gondii*. In addition, we found components in chromerid algae closely related to apicomplexans, e.g. MCU, MICU and IP3R/RYR are present in *Vitrella brassicaforma*, and MCU and MICU are found in *Chromera velia* (Fig. 2E). These data demonstrate that genes involved in mitochondrial Ca^2+^ transfer in mammals have been independently lost at least 19 times through evolution, including in apicomplexans.

### Depletion of VDAC leads to disruption of mitochondrial morphology

**Fig. 3.**
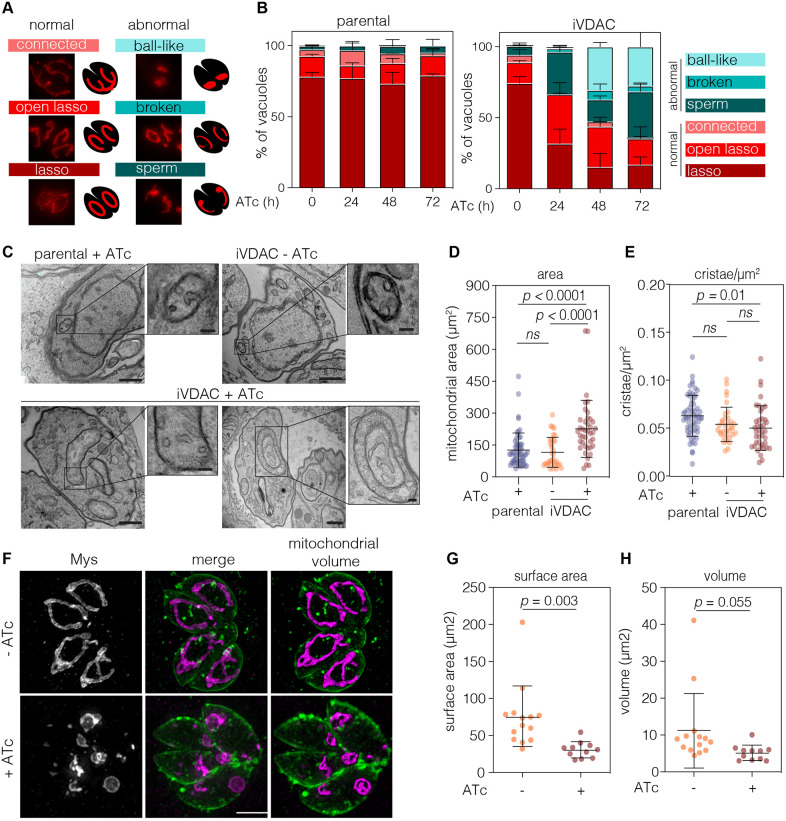
**Depletion of VDAC results in mitochondrial morphological defects.** (A) Mitochondrial morphology of intracellular parasites was scored as indicated. (B) Quantification of mitochondrial morphology from parental and iVDAC lines at indicated time points of ATc treatment. 100 vacuoles from two independent experiments were quantified; results are mean+s.d. No significant changes were seen in the parental lines upon ATc treatment at any points, but there were significantly fewer open lassos (*P*<0.0001) and significantly more sperm and collapsed morphologies (*P*<0.0006) in iVDAC at 24, 48 and 72 h post-ATc addition. *P*-values from two-way ANOVA with Tukey correction (C) Representative transmission electron microscopy (TEM) images of parental parasites and iVDAC parasites, untreated or treated with ATc for 48 h. Scale bars: 500 nm. Insets show detail of mitochondrion structures. Scale bars: 100 nm. (D,E) Quantification of mitochondrial area (D) and number of cristae/µm^2^ (E) from electron microscopy images. At least 30 parasites quantified per condition from *n*=2 independent experiments, results are mean±s.d., *P*-values from one-way ANOVA with Tukey correction. ns, no significant difference. (F) Super-resolution structured illumination microscopy (SR-SIM) projections of mitochondrion (Mys; magenta) and plasma membrane (SAG1; green) of iVDAC parasites, untreated and treated with ATc for 48 h. Volume projections of mitochondria are shown, in which substantial changes in mitochondrial morphology can be seen. Scale bar: 5 µm. (G,H) Quantification of mitochondrial surface area (G) and volume (H) calculated from volume projections, of at least ten vacuoles containing two parasites. Results are mean±s.d., *P*-values from unpaired Student′s *t*-test.

It has been shown that disruption of VDAC can cause changes in mitochondrial morphology (Ferecatu et al., 2018; Park et al., 2010). Previously, we defined three main mitochondrial morphologies detected by immunofluorescence microscopy in wild-type intracellular *Toxoplasma* tachyzoites (Ovciarikova et al., 2017). VDAC depletion resulted in three additional morphologies, which we named connected (for mitochondria that are connected between a number of parasites), broken and ball shaped, and which we scored from microscopy images (Fig. 3A). Addition of ATc to the parental line did not affect mitochondrial morphology, which presented as mostly lasso or open lasso shapes, as previously reported (Lacombe et al., 2019; Ovciarikova et al., 2017) (Fig. 3B). However, treatment of the iVDAC parasite line revealed a significant increase in abnormal morphologies at 24, 48 and 72 h post-ATc addition (Fig. 3B). *T. gondii* mitochondrial morphology is also known to change upon treatment with the anti-parasitic drug monensin (Lavine and Arrizabalaga, 2012). However, there was no change in the sensitivity to monensin between untreated and treated iVDAC parasites (Fig. S4), suggesting that disruption of mitochondria morphology through lack of VDAC did not sensitise the parasites to further drug-induced disruption.

To investigate the morphology of the mitochondrion in more detail, transmission electron microscopy (TEM) was used. In agreement with the above observation, there were no apparent effects of ATc on the parental mitochondria. In contrast, at 48 h post-ATc treatment, the iVDAC mitochondrion appeared larger and occasionally contained vesicular structures (Fig. 3C). Quantification of mitochondrial area from TEM images demonstrated that, in the sections imaged, the regions of mitochondria had significantly greater area in the iVDAC line after ATc treatment (Fig. 3D). VDAC depletion has been shown to affect cristae morphology in mouse muscle fibre (Baghel and Thakur, 2019). Previously, depletion of mitochondrial proteins in *T. gondii* resulted in a change in the density of mitochondrial cristae (Huet et al., 2018; Mühleip et al., 2021); however, depletion of VDAC in *T. gondii* did not change the density of cristae within the mitochondria (Fig. 3E).

TEM sections were selected based on the presence of mitochondria and so do not represent the total mitochondrial volume of the parasites. To determine how the total volume of a mitochondrion changed upon depletion of VDAC, we quantified the surface area and total volume of mitochondria per parasite, using super-resolution structured illumination microscopy (SR-SIM). *Z*-stacks of vacuoles containing two to four iVDAC parasites at 48 h post-ATc addition stained with anti-Mys (mitochondrion) and anti-SAG1 (parasite plasma membrane) antibodies were collected (Fig. 3F). The volume and surface area of mitochondria were automatically quantified from 3D-rendered projections of the anti-Mys signal. Depletion of VDAC resulted in a 59% decrease in mitochondrion surface area (Fig. 3G) and a 55% decrease in mitochondrion volume at 48 h (Fig. 3H). These results demonstrate that loss of VDAC results in a significant alteration in gross mitochondrial morphology.

### VDAC depletion leads to alterations in mitochondrial metabolism and protein import

**Fig. 4.**
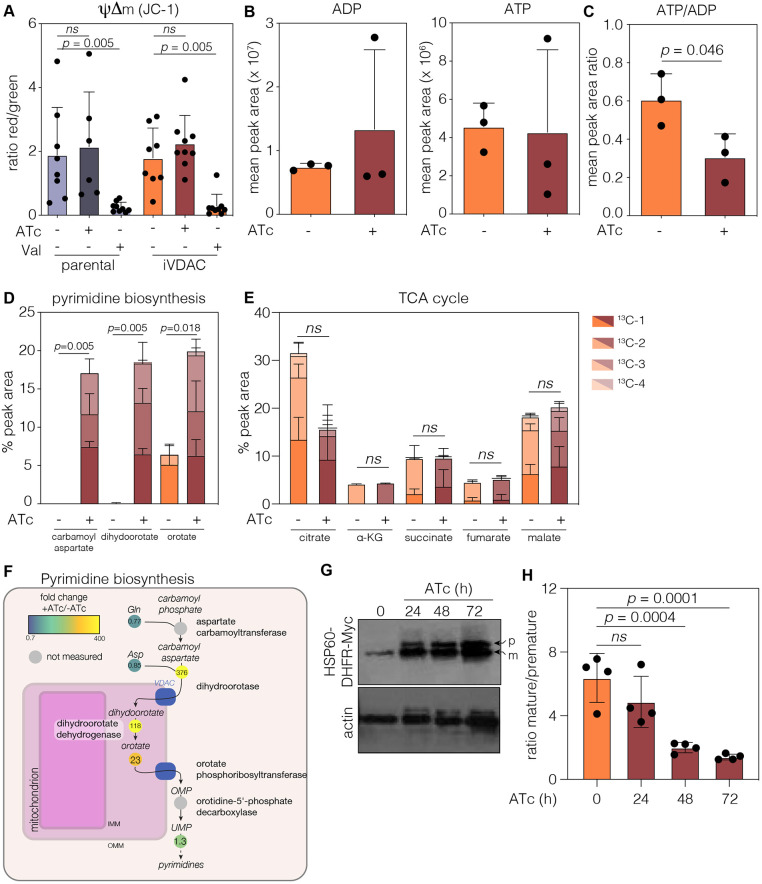
**Mitochondrial functions are mildly affected by depletion of VDAC.** (A) Mitochondrial membrane potential (ΔΨm) quantified using JC-1 probe. Results show mean ratio of red/green fluorescence events+s.d., as recorded by flow cytometry. Valinomycin (Val) was included as a membrane potential depolarising control. *P*-values from unpaired Student's *t*-test (*n*=at least five independent experiments). ns, no significant difference. (B) Quantification of ADP and ATP levels from labelled metabolomics. Values are combination of labelled and unlabelled peak areas. Points are mean of the peak recorded area+s.d., *n*=3. (C) Ratio between ADP and ATP levels, *P*-values from unpaired Student's *t*-test, *n=*3. (D,E) Quantification of selected labelled metabolites in the pyrimidine biosynthesis pathway (D) and the TCA cycle (E) from iVDAC parasites untreated and at 48 h post-ATc addition. Carbamoyl aspartate and dihydroorotate were almost undetectable in untreated parasites. Bars represent mean+s.d. of each species of labelled metabolite, *n*=3. (F) *T. gondii* pyrimidine pathway showing fold change in metabolite levels upon treatment with ATc, along with the predicted localisation of key enzymes in the pathway. Dihydroorotate dehydrogenase (DHODH) is found on the outer face of the inner mitochondrial membrane. (G) Protein import assays were performed after transient transfection of the iVDAC line with HSP60L-DHFR-Myc and treatment with ATc for the indicated time. Premature (p) and mature (m) bands can be identified by western blotting, with actin used as loading control. Accumulation of premature form can be seen as early as 24 h post-ATc treatment. Representative of *n*=4 experiments. (H) Densitometry obtained from bands shown in G regarding mature/premature ratio after ATc treatments. Results are mean±s.d., *P*-values from one-way ANOVA with Dunnett's correction, *n*=4.

Owing to the growth defect and the significant alterations in mitochondrial morphology, we wanted to determine whether mitochondrial physiology was altered upon VDAC depletion. Parasite mitochondrial membrane potential (ΔΨm) was assessed using flow cytometry of parasites stained with the fluorescent probe JC-1 (Fig. 4A) as previously described (Brooks et al., 2010; Maclean et al., 2021; Mühleip et al., 2021). As expected, treatment with valinomycin (Val) resulted in a decrease in the ratio of red to green fluorescence, indicating a loss of membrane potential (Brooks et al., 2010; Maclean et al., 2021). In contrast, depletion of VDAC for 48 h did not result in a significant change in ΔΨm (Fig. 4A).

In mammalian cells, VDAC is the major ATP and ADP transporter across the mitochondrial outer membrane, and depletion of VDAC isoforms results in a decrease in the cellular ATP/ADP ratio (Krammer et al., 2015; Maldonado et al., 2013). Untreated and 48 h ATc-treated iVDAC parasites were labelled with isotope-labelled ^13^C-U-D-glucose, and total ATP and ADP relative levels were quantified by liquid chromatography–mass spectrometry (LC-MS) (Fig. 4B). No significant changes in ADP or ATP levels were seen upon VDAC depletion; however, we did observe a small (*P*=0.046, unpaired Student's *t*-test) decrease in the ATP/ADP ratio (Fig. 4C), suggesting that VDAC may have a role in nucleotide transport in *T. gondii*, although it is possible that the residual VDAC expression present allows for some level of nucleotide transport.

In addition to ATP/ADP, VDAC is responsible for transportation of numerous small metabolites across the mitochondrial outer membrane (Hodge and Colombini, 1997; Lee et al., 2011; Pusnik et al., 2009). To determine how VDAC depletion affected global parasite metabolism, we analysed the ^13^C-U-D-glucose-labelled metabolites further. We observed a dramatic accumulation of carbomyl aspartate, dihydroorotate and orotate (Fig. 4D) after VDAC depletion. These metabolites are intermediates in the pyrimidine biosynthesis pathway (Fig. 4F). Interestingly, both carbomyl aspartate and dihydroorotate were undetectable in the untreated iVDAC line, while orotate was present at a low level. We did not see changes in the levels of pyrimidines upon VDAC depletion (results not shown); however, *T. gondii* is known to have a pyrimidine salvage pathway (Donald and Roos, 1995), which might explain the steady pyrimidine level. These results suggest that depletion of VDAC leads to dysregulation of the pyrimidine biosynthesis pathway. To confirm that addition of ATc did not cause a global change in mitochondrial metabolism, we also examined levels of the tricarboxylic acid (TCA) cycle intermediates citrate, α-ketoglutarate, succinate, fumarate and malate, which are expected to have specialised transporters. We saw no significant changes in levels upon ATc treatment (Fig. 4E), suggesting that addition of ATc did not induce global changes to mitochondrial metabolism.

Beyond small molecules, VDAC has also been shown to have a role in mitochondrial protein import in both plants and yeast through interacting with components of the translocon machinery (Ellenrieder et al., 2019; Salinas et al., 2006). To assess whether depletion of VDAC affected mitochondrial protein import in *T. gondii*, we assessed the maturation of the mitochondrial-targeted marker HSP60L-DHFR-Myc (van Dooren et al., 2016) through its transient expression in iVDAC parasites (Fig. 4G). A similar technique has been used successfully to assess protein import into the apicoplast and mitochondria in *T. gondii* (Sheiner et al., 2015, 2011; van Dooren et al., 2016). Depletion of VDAC caused a significant accumulation of the premature form of the protein by 48 h post-ATc addition (Fig. 4H), suggesting that protein import into the mitochondrion is disrupted by depletion of VDAC.

### Putative mitochondrion–ER contact sites are reduced upon VDAC depletion

**Fig. 5.**
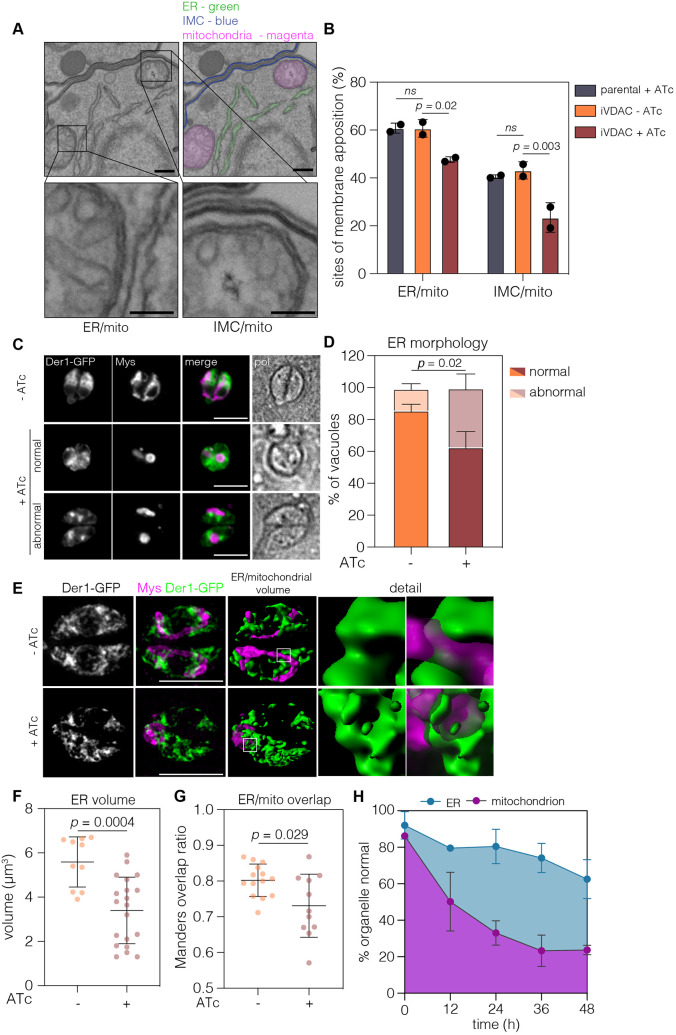
**Depletion of VDAC leads to changes in ER morphology and reduction of mitochondria–endoplasmic reticulum (ER) contacts.** (A) Representative TEM of putative ER–mitochondria and proposed IMC–mitochondria membrane contact sites from parental parasites. Panel with relevant membranes indicated in pseudocolour: IMC, blue; mitochondria, magenta; ER, green. Scale bars: 500 nm, 100 nm (insets). (B) The percentage of areas of membrane apposition from parental and iVDAC parasites with or without ATc treatment. At least 88 sections quantified per condition. *P*-values from two-way ANOVA with Tukey’s multiple comparisons. ns, no significant difference. (C) iVDAC parasites transiently expressing the ER marker Der1-GFP were ATc treated for 48 h and fixed, and ER morphology was examined. Upon VDAC depletion, the ER of many parasites lost its normal morphology and rounded up into small foci within the parasite. Scale bars: 5 µm. (D) Quantification of ER morphology, scored as normal or abnormal. Results are mean+s.d., *n*=3. Significant increase in abnormal morphology after treatment, *P*=0.019, unpaired Student's *t*-test. (E) Representative 3D reconstructions of ER (Der1-GFP, green) and mitochondria (Mys, magenta) in iVDAC parasites at 48 h post-ATc addition. Scale bars: 5 µm. (F) The volume of parasite ER was calculated from untreated and treated parasites. Points represent individual parasite ER with lines at mean, ±s.d. Significance from unpaired Student's *t-*test. (G) The ratio of overlap of the ER (Der1-GFP) with the mitochondria (anti-Mys) was quantified from SR-SIM images. Plots are mean with whiskers at 10–90%. Significance from Welch’s *t*-test, ratios calculated from at least 11 vacuoles. (H) Quantification of organelle morphology at selected time points post-ATc addition. Results are mean±s.d., *n*=3 from at least 70 vacuoles/condition.

In mammalian cells, VDAC proteins, along with ER and cytosolic components, mediate sites of mitochondrial–ER juxtaposition, suggested to be membrane contact sites (MCS) (Szabadkai et al., 2006). To determine whether similar sites of membrane apposition might be present between the mitochondrion and the ER in *T. gondii*, we analysed random thin sections of the parental parasite line. Morphologically, we defined membrane apposition as a constant distance of less than 30 nm between the two organelles over stretches of at least 100 nm (Ovciarikova et al., 2017). Using these parameters, putative contacts between the mitochondria and the IMC were seen, as previously reported (Jacobs et al., 2020; Ovciarikova et al., 2017), as well as apposition between the mitochondria and ER (Fig. 5A), implying that close contact between these organelles is frequent in *T. gondii*. Sections of parental and iVDAC parasites, treated and untreated with ATc, revealed that ∼61% of parental parasites exhibited membrane apposition between the mitochondrion and the ER, while 43% has contact between the IMC and mitochondrion. Upon depletion of VDAC, contacts between the ER and mitochondrion, and IMC and mitochondrion were significantly reduced (Fig. 5B). Thus, VDAC depletion results in reduction of mitochondrial contacts with both the IMC and the ER.

To examine how the endoplasmic reticulum was affected by VDAC depletion, iVDAC parasites were transiently transfected with a construct expressing the ER-resident Derlin1 (Der1) fused to GFP (Agrawal et al., 2009). We found that depletion of VDAC for 48 h resulted in significant changes in the localisation and distribution of the ER (Fig. 5C). In the absence of VDAC, the ER morphology changed from a perinuclear, reticular network found throughout most of the cytoplasm in untreated parasites, to the accumulation of Der1-GFP in foci and loss of the spread, perinuclear distribution. Forty-eight per cent of VDAC-depleted parasites displayed this abnormal ER localisation, as opposed to only 13% of the untreated parasites (Fig. 5D).

On the basis of the profound effect of VDAC depletion on mitochondrial and ER morphology, we examined other parasitic organelles. However, we saw no changes in the parasite pellicles (Fig. S5A), the secretory micronemes (Fig. S5B) and rhoptries (Fig. S5C), or the parasite's plastid (apicoplast) (Fig. S5D) at 48 h post-ATc treatment.

To investigate the effect of VDAC depletion on the ER in more detail, we acquired SR-SIM images of iVDAC parasites expressing Der1-GFP (Fig. 5E). Upon depletion of VDAC, we observed a significant decrease in the volume of parasite ER signal in reconstructed 3D projections (Fig. 5F). We also observed a decrease in the proportion of ER/mitochondrial fluorescence signal overlap (Fig. 5G). These results suggest that the defects in the mitochondrial morphology coincide with defects in the distribution of the ER, and with the frequency of contacts between the mitochondrion and ER. We then quantified the proportion of abnormal mitochondria and ER in the same parasites over time (Fig. 5H). We saw that the defects in mitochondrial morphology appeared prior to changes in the ER, with about half the mitochondria appearing abnormal as soon as 12 h after ATc addition (*P*=0.006, one-way ANOVA with Dunnett's correction), while significant changes in the ER are then seen between 36 h and 48 h (*P*=0.004, one-way ANOVA with Dunnett's correction). These temporal effects suggest that the alterations in ER morphology are in response to the changes in mitochondrial morphology, and the unchanged organelles testify to specificity of this outcome.

Taken together, these results suggest that MCS may be present between the mitochondrion and ER, and that depletion of VDAC leads to both a decrease in MCS and a specific change in the morphology of the ER.

## DISCUSSION

Here, we describe a *T. gondii* homologue for the ubiquitous mitochondrial porin, VDAC. As the most abundant pore in the outer mitochondrial membrane across phyla, VDAC performs an important role in allowing passage of macromolecules and metabolites between the cytosol and intermembrane space. In *T. gondii*, depletion of VDAC resulted in subtle changes in metabolite abundance, and has no significant effect on the ΔΨm or Ca^2+^ homeostasis. Instead, dramatic changes in mitochondrial and ER morphology were observed, in concert with reduced ER–mitochondrial contacts, as well as impaired parasite replication, leading to a growth defect. These results demonstrate the conserved roles of VDAC across eukaryotes, while highlighting some important changes in the Apicomplexa.

One of the canonical roles of VDAC is as a pore for nucleotide transport across the outer mitochondrial membrane. In several systems, depletion of VDAC leads to a decrease in the ATP/ADP ratio (Maldonado et al., 2013; Maldonado and Lemasters, 2014) owing to inhibition of the flow of ATP and ADP across the mitochondrial membrane. In *T. gondii*, we see a similar phenotype; although absolute levels of cellular ATP and ADP do not differ substantially, the ratio of ATP/ADP decreases, which suggests a role for VDAC in mediating nucleotide transport across the mitochondrion in these parasites*.* Given that ∼20% VDAC expression is maintained under ATc (even as much as 5 days post-ATc addition), it is possible that the residual VDAC is able to perform some of this function under these conditions, thus explaining the relatively mild defect compared to other systems (Maldonado et al., 2013). VDAC also acts as a pore for other metabolites, including amino acids and other metabolic intermediates (Ellenrieder et al., 2019; Hodge and Colombini, 1997; Pusnik et al., 2009). Interestingly, we found that depletion of VDAC leads to a large accumulation of intermediates in the pyrimidine biosynthesis pathway. Pyrimidine synthesis occurs in the cytosol, with the exception of the conversion of dihydroorotate to orotate, catalysed by dihydroorotate dehydrogenase (DHODH), which is localised to the inner mitochondrial membrane in *T. gondii* (Hortua Triana et al., 2012). In *Plasmodium*, chemical inhibition of DHODH leads to accumulation of carbamoyl aspartate and dihydroorotate (Creek et al., 2016). We suggest that dihydroorotate, and possibly orotate, requires transport via VDAC across the outer mitochondrial membrane, and, upon the depletion of VDAC, intermediates in the pathway accumulate. Depletion of VDAC does not appear to affect the function of the TCA cycle, which relies on specialised solute transporters (van Dooren et al., 2006), demonstrating that the effect of VDAC depletion is specific to the pyrimidine pathway. The pyrimidine pathway is of major interest in *Plasmodium*, for which DHODH has emerged as an important drug target (Hoelz et al., 2018; Painter et al., 2007), and this potential role of VDAC in apicomplexan parasites will merit further study.

VDAC has been shown to be linked to protein import across the outer mitochondrial membrane in yeast and plants (Ellenrieder et al., 2019; Salinas et al., 2006). In *T. gondii*, depletion of VDAC led to a defect in the import of a mitochondrial localised HSP60L, previously used to demonstrate protein import defect upon depletion of components of the TOM complex (van Dooren et al., 2016). Interestingly, it was previously suggested that, although the TOM complex is essential for the parasite, some protein import could occur in its absence (van Dooren et al., 2016). It is possible that VDAC is involved in this bypass. However, VDAC depletion also leads to morphological collapse of the mitochondrion, and this change in morphology may indirectly inhibit protein import.

In plant cells, VDAC is also thought to be required for import of tRNAs (Salinas et al., 2014, 2006). The mitochondrial genome of *T. gondii* is severely reduced and encodes only subunits of the electron transport chain and rRNAs, which require tRNA import for translation (Esseiva et al., 2004; Lacombe et al., 2019; Namasivayam et al., 2021; Pino et al., 2010). However, we do not see any changes in the membrane potential of the mitochondria upon VDAC depletion using the fluorescent probe JC-1. Although there are some drawbacks to JC-1, including its activity as a photosensitiser and some variability in loading efficiency (Zorova et al., 2018), in *T. gondii* it has proved useful in assessing membrane potential upon changes in ATP synthase complex formation (Mühleip et al., 2021), as an indirect measure of cellular viability (Brooks et al., 2010), and in detecting membrane potential defect under depletion of complex III subunits (Maclean et al., 2021). The lack of significant change in ΔΨm provides indirect evidence that the electron transport chain remains functional, thus likely no defect in tRNA import occurs. This is in agreement with results from *Trypanosoma*, which does not require VDAC for tRNA import (Pusnik et al., 2009). The mechanism for tRNA import into the mitochondrion of *T. gondii* remains unknown, and our observation may highlight the diverse strategies evolved to fulfil this essential function, while it cannot be excluded that the residual 20% of VDAC suffice for tRNA import.

In *T. gondii*, close apposition of organelles has been noted, including between the mitochondrion and IMC (Jacobs et al., 2020; Ovciarikova et al., 2017), the ER and the apicoplast (Tomova et al., 2009), and the mitochondrion and apicoplast (Kobayashi et al., 2007; Nishi et al., 2008); however, the molecular identity of many of the players is only beginning to be understood (Jacobs et al., 2020). Based on our observation of TEM images of wild-type parasites, the mitochondrion of *T. gondii* appears to form frequent, close associations with the ER. VDAC is a natural candidate to mediate this interaction, based on results from other organisms, and, in *T. gondii*, depletion of VDAC leads to significant and specific changes in both mitochondrion and ER morphology. This is in contrast to LMF1, a mitochondrial protein likely involved in mitochondria–IMC contacts (Jacobs et al., 2020), the deletion of which leads to a change in mitochondrial morphology, but no change in ER distribution (Jacobs et al., 2020). These results suggest that VDAC is involved in interactions between the mitochondrion and the ER, which are important to maintain the distribution of both organelles throughout the parasite. In mammalian cells, the distribution of organelles is also mediated and supported by the tubulin, and, to a lesser extent, actin cytoskeletons (Barlan and Gelfand, 2010; Frederick and Shaw, 2007). However, in *T. gondii*, the tubulin cytoskeleton is involved in maintaining cell shape but does not pass through the body of the cell (Morrissette and Sibley, 2002), and alterations in parasite actin do not appear to affect the mitochondrion or ER morphology (Tosetti et al., 2019). This may suggest that MCS in *T. gondii* have a more important role in maintaining organelle distribution than in other species, as suggested upon the mitochondrial–IMC contacts description (Ovciarikova et al., 2017). Given the timing of the defect observed – mitochondrial morphology defect, followed by ER morphology defect and then a replication defect – we propose that VDAC-mediated ER–mitochondrial contacts are important for parasite replication. Combined with the precisely timed and sequential division of organelles during *T. gondii* endodyogeny (Kobayashi et al., 2007; Nishi et al., 2008), our observations raise the possibility that ER–mitochondrial contacts may play a role in this tightly controlled process.

The mammalian contact involving VDAC, GRP75 (also known as HSPA9) and IP3R (also known as ITPR) interacts with MCU and MICU to regulate Ca^2+^ mobilisation. Our phylogenetic profiling confirmed the genes coding for MCU, MICU and IP3R as ancestral eukaryotic genes. Each major lineage evolved from an ancestor with a full complement of these proteins; however, we report no fewer than 19 independent losses of all three components (Fig. S3). The co-occurrence of these proteins in many lineages is suggestive of their functional overlap, potentially as mediators of Ca^2+^ homeostasis as proposed for mammals. Although these proteins are ancestral and critical components of eukaryotes, our data suggest that either (1) compensatory mechanisms exist that can take the role of this system when lost, (2) the system can be readily replaced by a novel mechanism through functional innovation or horizontal gene transfer, or (3) the well-studied functions of these proteins in animals represent a lineage-specific adaptation. From our data on VDAC we cannot distinguish these options; however, future work on determining the composition of mitochondrial/ER contacts in Apicomplexa may help to answer these questions.

In summary, our results give the first indication that the broadly conserved outer mitochondrial porin, VDAC, is present and functional in *T. gondii*. VDAC appears to have a role in protein and metabolite transfer, and is required to maintain the morphology of both the mitochondria and the ER. The study of membrane contact sites in the context of this divergent parasite is a new area with likely important implications in regulation of organelle function during the lytic lifecycle. Our observed multiple losses of components of the mammalian Ca^2+^ homeostasis contact, along with the finding that VDAC depletion does not lead to defect in processes that are affected by Ca^2+^ changes, highlight divergence between parasite and host in this critical pathway.

## MATERIALS AND METHODS

### Parasite culture and genetic manipulation for line generation

*T. gondii* parasites were grown in human foreskin fibroblast (HFF) cells in supplemented Dulbecco's modified Eagle medium (DMEM) supplemented with penicillin/streptomycin with 10% foetal bovine serum (FBS) (Gibco). Parasites were incubated at 37°C with 5% CO_2_ and 100% humidity.

For transfections, electroporation was performed using 1×10^7^ freshly egressed parasites using a Bio-Rad electroporator following the manufacturer's instructions. To localise tagged Myc-VDAC, parental TATiΔku80 (Sheiner et al., 2011; Jacot et al., 2014) parasites were transfected with a plasmid containing a pTUB8 promoter and VDAC cDNA coding sequence fused with an N-terminal Myc epitope tag, amplified using primers P1 and P2 (Table S1), named pTUB8_Myc_VDAC.

To create the iVDAC parasite line, the promoter replacement strategy was used following a strategy previously described (Jacot et al., 2014; Sheiner et al., 2011). The ChopChop tool was used to identify the best gRNA containing the ATG of the gene (http://chopchop.cbu.uib.no/), and primers P3 and P4 were duplexed. gRNAs were cloned into a gRNA and Cas9 expression vector (Addgene #80636) using the BsaI restriction site. A PCR product containing the ATc-repressible promoter followed by DHFR selectable cassette was amplified by PCR from pDT7S4myc using primers in P5 and P6 (Table S1) (Jacot et al., 2014; Sheiner et al., 2011). Parasites were co-transfected with 50 μg of the gRNA/CAS9 vector and the purified PCR products into the TATiΔku80 parental line. Cassette integration was selected for using 1 µM pyrimethamine for 3 weeks. After cloning into 96-well plates, clones were PCR screened using primers P7 and P8 (5′ integration) and P9 and P10 (3′ integration) (see Table S1) and sequenced.

To generate the tdTom/iVDAC line, pCTR2T-TGME49_215430-tomato [tandem tomato (2t) expression cassette] (van Dooren et al., 2016) transgene was introduced into the iVDAC line, followed by enrichment of the stably expressing fluorescence parasite population by cell sorting. Clones of this line were isolated by serial dilution into 96-well plates and fluorescent clones selected.

### C-terminal triple HA epitope tagging of VDAC

The ChopChop tool was used to identify gRNA targeting the stop codon, which was cloned (primers 13/14; Table S1) into a U6 promoter and CAS9-GFP-expressing vector (Tub-Cas9YFP-pU6-ccdB-tracrRNA) using the BsaI restriction site. The CAT selection cassette and triple HA epitope were amplified by PCR from a p3HA.LIC.CATΔpac plasmid (Sheiner et al., 2011). The gRNA/CAS9 vector-PCR product mixture was transfected into iVDAC line by electroporation, and cassette integration was selected with chloramphenicol. Positive clones were isolated by serial dilution and confirmed by PCR analysis. Integration was tested with immunofluorescence.

In all experiments, 0.5 µM ATc was added to the iVDAC line to repress the regulatable promoter.

### Identification of TgVDAC and phylogenetic analyses

The bioinformatics tool tBLASTn was used to compare amino acid sequences against the translated nucleotide *T. gondii* database to find a potential candidate. For the phylogenetic tree, the evolutionary history was inferred using the Neighbor-Joining method (Saitou and Nei, 1987). The optimal tree with the sum of branch length=14.07947748 is shown. The tree is drawn to scale, with branch lengths in the same units as those of the evolutionary distances used to infer the phylogenetic tree. The evolutionary distances were computed using the Poisson correction method and are in the units of the number of amino acid substitutions per site. The analysis involved 15 amino acid sequences. All positions containing gaps and missing data were eliminated. There were 226 positions in the final dataset. Evolutionary analyses were conducted in MEGA6 (Tamura et al., 2013).

### Immunofluorescence assay

Parasites were inoculated onto 24-well plates containing HFF cells on coverslips for the different time points, washed with phosphate buffered saline (PBS), and cells were fixed in 4% paraformaldehyde for 20 min at room temperature (RT). After three PBS washes, cell membranes were permeabilised and sample blocked by incubation with 0.2% Triton X-100/PBS (PBST) and 2% bovine serum albumin (BSA) for 20 min at RT. Cells were then incubated for 1 h with the correspondent primary antibodies at RT in a wet chamber. Primary antibodies were diluted in 1% BSA in PBS-Triton (Table S2). Cells were washed three times with PBST and incubated with the correspondent secondary antibody (diluted in 1% BSA in PBST) (goat anti-mouse or anti-rabbit conjugated to AlexaFluor 594 or 488, Invitrogen; 1:1000) for 1 h at RT in the dark. Following final washes, nuclei were stained by addition of 1 µg/ml 4′,6-diamidino-2-phenylindole dihydrochloride (DAPI; Sigma-Aldrich) and coverslips mounted onto glass slides using FluoromountG (Southern Biotech). Images were acquired using a DeltaVision Core microscope (AppliedPrecision, GE Healthcare) as described (Ovciarikova et al., 2017) and were analysed using FIJI ImageJ 64 software (Schindelin et al., 2015).

### RT-qPCR

For testing downregulation of the *VDAC* gene upon ATc addition at different time points, RT-qPCR was performed as previously described (Biddau et al., 2018). Total RNA was isolated from freshly egressed treated parasites using TRIzol^TM^ (Invitrogen, Thermo Fisher Scientific) following the manufacturer's instructions. To remove DNA contamination in the sample, RNA was treated with RNase-free DNase (Invitrogen, Thermo Fisher Scientific). Then, 0.5 µg of total RNA was used to synthesise cDNA using a SuperScript^®^ VILO™ cDNA Synthesis Kit (Invitrogen, Thermo Fisher Scientific) following the manufacturer's instructions. Quantitative PCR was completed on a real-time PCR instrument (7500 Fast Real-Time PCR System, Applied Biosystems) with the PowerUP^TM^ SYBR^TM^ Green Master Mix from Applied Biosystems using primers P11 and P12 (Table S1).

### Western blotting

We collected 2×10^6^ of filtered (3 μm) parasites per sample. Pellets were resuspended in 75% water and 25% 4× loading dye (1 ml of 1 M Tris-HCl pH 6.8, 0.4 g SDS, 2 ml glycerol, 1 ml 2-mercaptoethanol, 0.01 g Bromophenol Blue, dH_2_O up to 5 ml) and boiled at 95°C for 5 min. Samples were run on 10% acrylamide gels and transferred using a semi-dry method onto nitrocellulose membrane (Whatman). Membrane was blocked with blocking buffer (5% milk, PBS/0.2% Tween 20) for 1 h at RT. Primary antibody was added in blocking buffer and membrane incubated for 1 h at RT, followed by 3×5 min washes with washing buffer (PBS/0.1% Tween 20). Secondary antibody was added in blocking buffer and membrane incubated for 1 h at RT, followed by 3×5 min washes with washing buffer (PBS/0.2% Tween 20). Signal was developed with Pierce ECL Western Blotting Substrate.

### Parasite growth assay

Freshly egressed tdTom/iVDAC parasites were filtered through a 3-μm polycarbonate filter, centrifuged and resuspended in parasite culture medium without Phenol Red (Gibco BRL Life Technologies, Rockville, MD). Five hundred parasites/well were used to infect confluent HFF cells in black optical bottom 96-well culture plates in triplicate. Parasites were treated (or not) with 0.5 µM ATc, fluorescence was read daily in a BMG Fluostar plate reader with constant gain, and data from the three wells were averaged. Blank wells, containing media and host cells with ATc, were measured simultaneously on the same experimental plate. Parasite growth was evaluated by increase in fluorescence over time, normalised to day 0.

### Mitochondrial potential using JC-1

Freshly lysed parasites were 3.0 µm-filter purified and incubated with 1.5 µM JC-1 (Thermo Fisher Scientific) for 15 min at 37°C. After incubation, parasites were washed three times by centrifugation and re-suspended in DMEM without Phenol Red (Gibco BRL Life Technologies). Treatment with 10 µM Val for 10 min was included as a depolarising control. Unstained controls were used to define gates for analysis. Fifty thousand events per treatment were collected on a BD FACSCalibur^TM^ (Becton Dickinson, San Jose, CA) flow cytometer, and data were analysed using BD CellQuestPro for changes in the ratio of green to red, as an indicator of fluctuations in membrane potential.

### DuoLink PLA assay

Parasites were seeded onto confluent HFF monolayer grown on glass coverslips. Cells were fixed with 4% paraformaldehyde for 20 min at RT. The cells were permeabilised by incubation with PBS/0.2% Triton X-100 for 20 min at RT. DuoLink assay (Sigma-Aldrich, DUO92101-1KT) was carried out according to the manufacturer's instructions with the following modification: washing steps were extended as 3× wash with 1 ml washing buffer per coverslip.

### Protein import assay

Parasites were transfected with Hsp60L-mDHFR-cMyc (van Dooren et al., 2016) (a kind gift from Giel van Dooren, Research School of Biology ANU College of Science, Canberra, Australia), 24 h prior to the desired time point after growth in the presence or absence of 0.5 µM ATc. Freshly egressed extracellular parasites were filtered, collected by centrifugation (1500 ***g***, 10 min) and washed twice with PBS, and then pellets were lysed in 800 µl ice-cold lysis buffer NuPAGE LDS (Invitrogen) with 200 µl methanol and 2% β-mercaptoethanol. Samples were boiled for 5 min at 95°C. Ten micrograms of total protein, quantified using a NanoDrop™ spectrophotometer (Thermo Fisher Scientific), were separated on 12% SDS-PAGE gel and transferred (transfer buffer: 0.025 M Tris, 0.192 M glycine, 10% methanol) to a nitrocellulose membrane (0.45 μm, Protran™) for 60 min at 100 V. To control for protein transfer, the membrane was stained with Ponceau S red-staining solution (Sigma-Aldrich) after transfer. The membrane was then blocked with 5% BSA in Tris-buffered saline (TBS; 20 mM Tris, 150 mM NaCl) and 0.05% Tween 20 (Sigma-Aldrich) and incubated with the primary antibodies (Table S2) overnight at 4°C in a wet chamber. After three washes with TBS, anti-mouse horseradish peroxidase (HRP)-conjugated secondary antibody (Promega; 1:10,000) was incubated at RT for 45 min with agitation. The signal was developed using Pierce^™^ ECL Western Blotting Substrate (Thermo Fisher Scientific). FIJI was used for analysis of band intensity, and the ratio between the mature and premature band intensity was quantified.

### Mitochondrial morphology analysis

Mitochondria were visualised by immunofluorescence as described above. Fifty vacuoles of each treatment were counted, and mitochondrial morphology was assessed according to six different mitochondrial morphology categories: (1) open lasso, (2) lasso, (3) connected, (4) sperm, (5) broken and (6) ball-like. Parental and iVDAC parasites were grown in the presence or absence of ATc 0.5 µM for 24, 48 and 72 h, and mitochondria were visualised by immunofluorescence using an anti-TgMys antibody, as described above. Experiments were performed in triplicate.

### Super-resolution fluorescent microscopy

For super-resolution structural illumination microscopy (SR-SIM), stacks of vacuoles containing two or four parasites (with increments of 0.1 µm in a total of 5 µm) were imaged in a Elyra PS.1 super-resolution microscope (Zeiss, Jena, Germany) with a 63×/1.4 NA oil-immersion objective using ZEN black software (Zeiss). Five-phase SR-SIM images were reconstructed in the same software using the Structural Illumination manual processing tool. The 3D models were reconstructed in Imaris software (Oxford Instruments). The same software was used to calculate the volume and surface area of mitochondrial and ER signals. The Colocalization tool in the same software was used to calculate the overlap proportion (Meanders coefficient calculation) of mitochondrion:ER signals.

### TEM and morphometric analysis

LLC-MK_2_ cultures in 25 cm^2^ flasks were infected with tachyzoites of iVDAC mutant and incubated (or not) with 0.7 µM ATc for 72 h. After that, infected cells were fixed with 2.5% glutaraldehyde in 0.1 M sodium cacodylate buffer (pH 7.4) and post-fixed for 45 min in the dark in 1% osmium tetroxide, 1.25% potassium ferrocyanide and 5 mM CaCl_2_, in 0.1 M sodium cacodylate buffer (pH 7.4). After post-fixation, infected cells were en bloc stained with uranyl acetate and lead aspartate. Then, samples were dehydrated in acetone solutions of increasing concentrations (30–100%) and embedded in PolyBed 812 resin (Polyscience, Warrington, PA) using flat-embedding moulds (EMS, Hatfield, PA). Ultrathin sections (70–80 nm) from three different blocks of −ATc and +ATc iVDAC were obtained in a Leica UC6 ultramicrotome and collected in 400 mesh copper grids (two grids per block). Sections were observed in a JEOL 1200 EX and FEI Tecnai Spirit 120 transmission electron microscope, and obtained images were analysed using ImageJ software. Images were acquired at Centro Nacional de Biologia Estrutural e Bioimagem, Rio de Janeiro, Brazil.

### Gliding assay

Freshly egressed tachyzoites were 3.0 µm-filter purified, spun down and resuspended in Hank's balanced salt solution supplemented with 100 mM HEPES (HBSS-H), 20 mM EGTA at ∼5×10^6^ parasites/ml. All the assays were done in HBSS not containing Mg^2+^ or Ca^2+^, unless indicated. Parasites were layered onto poly-L-lysine-coated coverslips and allowed to adhere for 5–15 min at RT then incubated for 20 min at 37°C, in the presence or absence of 2 mM ionomycin (Santa Cruz Biotechnology, 56092-82-1 Ionomycin-3592). After incubation, parasites were fixed as above followed visualisation of gliding trails using α-SAG1 (Table S2) with no permeabilisation. Images were acquired using a DeltaVision Core microscope (AppliedPrecision, GE Healthcare) as described (Ovciarikova et al., 2017). Image brightness was adjusted using FIJI software to allow for clear visualisation of the trails, which were then traced with a wand tool, and the length of the trail was measured.

### Invasion assay

Parasite invasion was assessed using an invasion assay. First, 5×10^6^ artificially egressed parasites were centrifuged for 5 min at 500 ***g***, then resuspended in 200 µl volume and inoculated on confluent HFF cells on glass coverslips in a 24-well plate and incubated for 20 min at 37°C before washing with PBS and fixing. Extracellular parasites were stained using anti-SAG1 antibody (Table S2) in non-permeabilised cells. Subsequently, cells were permeabilised and stained with rabbit anti-GAP45 (Table S2) to visualise all parasites. A minimum of 200 parasites were counted from 20 fields per treatment, for each experiment, and the ratio of intracellular to extracellular parasites was calculated.

### Metabolomics extraction

Stable isotope labelling of intracellular *T. gondii* tachyzoites was done as described below with some modifications from previous protocols described for *T. gondii* (MacRae et al., 2012; Oppenheim et al., 2014). First, 2×10^8^ intracellular parasites were washed with glucose-free DMEM containing glutamine, FBS and antibiotics. That medium was immediately replaced with cold, glucose-free DMEM containing 4 mM ^13^C-U-D glucose (50:50). Parasites were incubated for 4 h under standard culture conditions and harvested. Both host cell and parasite metabolism was quenched by placing the culture flasks on ice. Cells were washed twice, and the parasites were scraped, syringed and filtered to remove host cell debris into ice-cold PBS. Parasites were pelleted at 4000 rpm, for 25 min at 4°C. After washing, the pellet was used to extract the sample in chloroform/methanol/water (1:3:1 v/v) buffer. After sonication for 2 min on ice, samples were incubated on ice under shaking for 1 h with sonication every 10 min. Samples were then centrifuged at 13,000 ***g*** for 10 min at 4°C. The supernatant was stored at −80°C prior to LC-MS analysis.

### LC-MS analyses

Metabolomics analyses were performed by LC-MS using an Ultimate 3000 LC system (Dionex, Camberley, UK) connected to a Q Exactive HF Hybrid Quadrupole-Orbitrap mass spectrometer (Thermo Fisher Scientific). The system was controlled by the software Chromeleon (Dionex) and Xcalibur (Thermo Fisher Scientific), acquiring both positive and negative ionisation mode. Chromatographic separation was performed with a ZIC-pHILIC chromatography column (150 mm×64.6 mm×65 mm; Sequant, Uemå, Sweden) using a two-solvent system consisting of solvent A (20 mM ammonium carbonate) and solvent B (acetonitrile). Table 1 shows the chromatographic conditions.

**Table 1.**
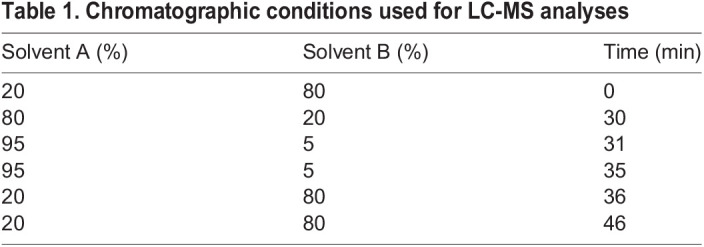
Chromatographic conditions used for LC-MS analyses

### Metabolomic data analysis

Vendor-specific raw data were initially centroided and converted into the open format mzXML for subsequent processing. PeakML files (Scheltema et al., 2011) were then generated by extracting the chromatographic peaks contained in the mzXML files using the detection algorithm from XCMS (Tautenhahn et al., 2008). The data-processing pipeline mzMatch.R (Jankevics et al., 2012) was used to sort and combine all PeakML files corresponding to replicates and to exclude all non-reproducible data. Further steps of noise filtering, gap filling and metabolite identification were performed on PeakML files utilising data obtained from metabolic standards run in parallel. For each metabolite of interest, the proportions of each isotopologue and its relative abundance in the sample were determined. The PeakML.Isotope.TargetedIsotopes function of mzMatch-ISO (Chokkathukalam et al., 2013) was used to scan the PeakML files for labelled metabolite quality and quantity.

### Statistical analysis

Data were analysed using GraphPad Prism software (version 5.00, La Jolla, CA). Unless otherwise indicated, analyses were performed using either Student's *t*-test or one-way ANOVA with correction for multiple comparisons as indicated.

### Cytosolic [Ca^2+^] quantification

GCaMP6 and mCherry-expressing plasmid (described in Stewart et al., 2017) was linearised with the uprt homology region and transfected into tachyzoites and selected with FUDR. Stable parasite populations were then treated with and without ATc, and GFP and mCherry fluorescence was measured using FACS LSR IIW as described in Uboldi et al. (2018). Briefly, A23187 and BIPPO was serially diluted at a 2× concentration. Basal tachyzoite fluorescence was measured, and equivolume of 2× agonist was added rapidly before further data acquisition for 2 min. All analysis was performed in FloJo v10.

### Homology searching and phylogenetic reconstruction

MCU, MICU, IP3R and RYR3 homologues were identified by using human sequences as BLAST queries into a subset of predicted proteomes from across the eukaryotic tree of life (Table S3) using the NCBI BLAST server, and the Joint Genome Institute's Mycocosm and Phycocosm databases (Grigoriev et al., 2014). Potential homologues were validated by a reciprocal BLAST into the *Homo sapiens* predicted proteome. When the original *H. sapiens* sequence was recovered, we concluded that an orthologue was identified. Validated orthologues were then used to identify extremely divergent orthologues in some lineages (e.g. apicomplexans). Accession numbers of retrieved sequences can be found in Table S3. To ensure that no highly divergent sequences were overlooked, we used our validated orthologue set to generate a Hidden Markov Model via the HMMer server at the European Bioinformatics Institute (Finn et al., 2011). We were unable to identify any additional candidate orthologues within our target set of species. We identified 19 instances in which MCU, MICU and IP3R were lost and mapped these losses onto a consensus tree of eukaryotes (Fig. S3).

## Supplementary Material

Supplementary information

Reviewer comments
