# Supplementary material for: Depletion of a Toxoplasma porin leads to defects in mitochondrial morphology and contacts with the endoplasmic reticulum
Source: J Cell Sci. 2021 Oct 20;134(20):jcs255299. doi: 10.1242/jcs.255299 (PMC8572010; doi:10.1242/jcs.255299)
Supplement: Supplementary information [file joces-134-255299-s1.pdf]

A

```
Tg      ----MVLFKDLNKSCADLLTKGYPH    21
Sc      --MSPPVYSDISRNINDLLNKDFYH    23
Hs      -MAVPPTYADLGKSARDVFTKGYGF    24
At      MVKGPGLYTEIGKKARDLLYRDYQG    25
               : ::.:.  *:: :.:
```

B

Species	Gene	Length (aa)	Homolgy to *T. gondii* VDAC (% identity)
T. gondii	TGME49_263300	290	n/a
H. hammondi	HHA_263300	290	99
N. caninum	NCLIV_024630	290	96
E. tenella	ETH_00023750	292	47
V. brassicaformis	Vbra_784	281	39
P. falciparum	PF3D7_1432100	289	29
T. brucei brucei	Tb927.2.2510	270	37
C. cayetanensis	cyc_02478	348	50
H. sapiens	VDAC2	294	33
A. thaliana	VDAC5	226	25
S. cervasiase	POR1	283	21
H. sapiens	VDAC3	283	26
H. sapiens	VDAC1	283	24

Fig. S1. Phylogeneti.alysis of VDAC from *T. gondii*

A) VDAC from *T. gondii* (Tg) contains glycine and lysine residues at positions 16 and 20 respectively which are conserved between human (Hs), yeast (Sc) and plants (At, D21 only). **B)** Table showing the gene ID, length (in amino acids) and percentage identity to *T. gondii* of VDACs from selected species.

Journal of Cell Science • Supplementary information

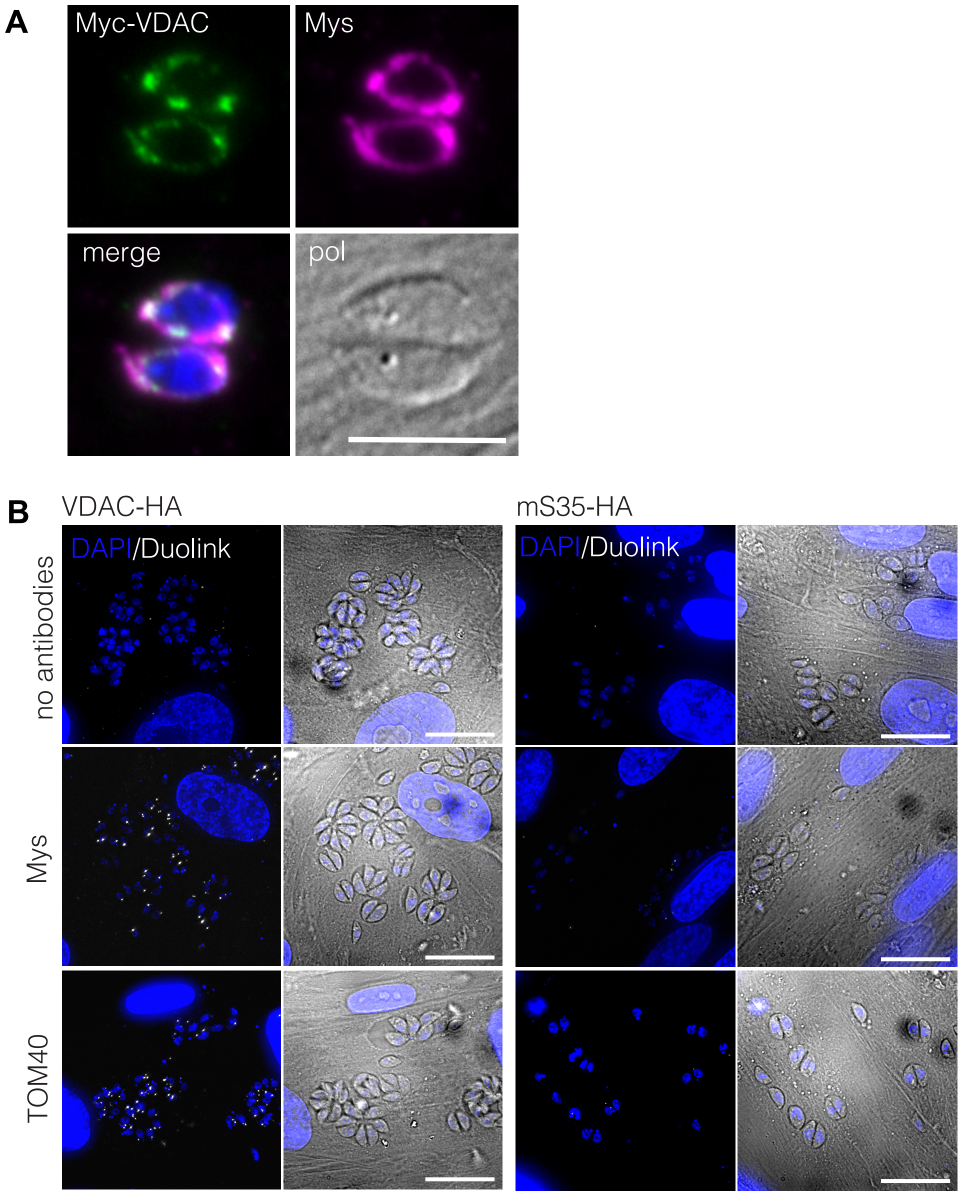

Figure S2. VDAC localises to the outer membrane of the mitochondrion
A) Overexpression of Myc-VDAC (green) co-localized with anti-Mys, a mito-chondrial membrane marker. Scale bar 5 μm. **B)** Larger scale representative images of the DuoLink PLA, showing many vacuoles per field, demonstrating that when seen, PLA signal is seen in most/all cells. Scale bar 20 μm

Journal of Cell Science • Supplementary information

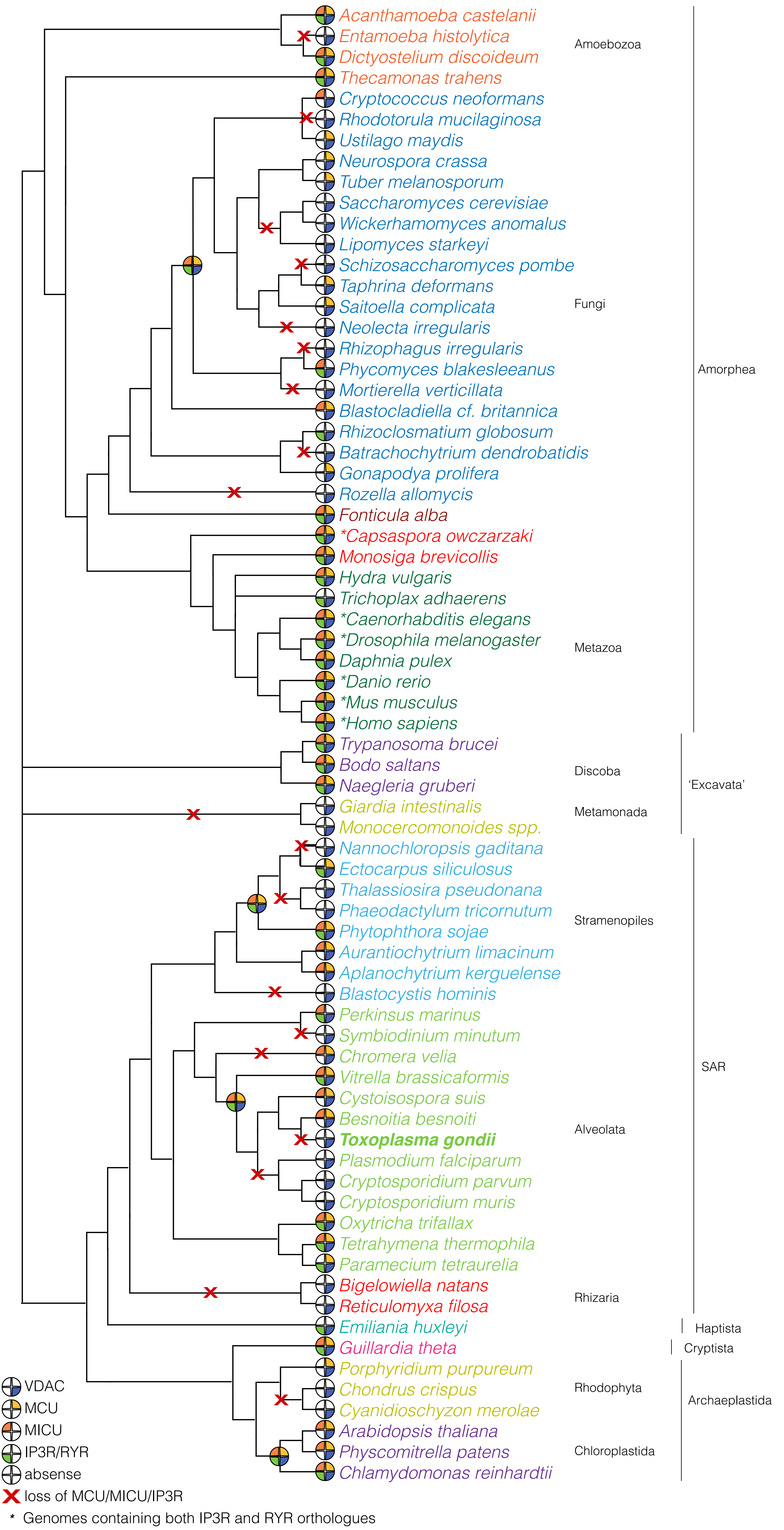

Journal of Cell Science • Supplementary information

Fig. S3. MCU, MICU, and IP3R have been lost 19 times in the evolution of eukaryotes
Orthologues of MCU, MICU, and IP3R were identified using BLAST and phylogenetic reconstructions. Organisms with identified orthologues are marked as indicated in the figure legend. Most eukaryotes contain homologues that could not be unambiguously identified as ITPR1 or RYR3. Aster-isks indicate organisms containing both ITPR1 and RYR3 orthologues.

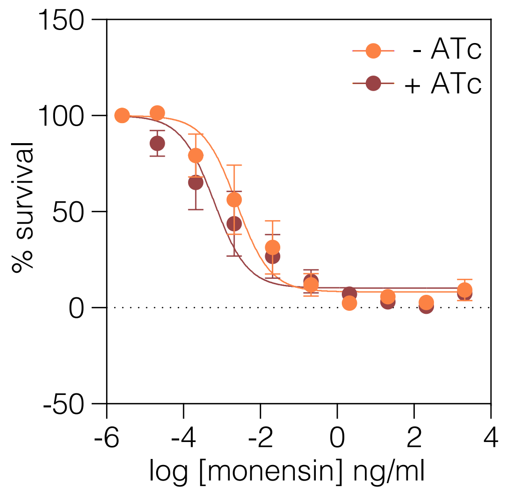

Fig. S4. Monensin IC50 is not affected by VDAC depletion
Growth curve generated using fluorescent iVDAC parasites grown with or without ATc in the prescenceof different concentrations of monensin. Results mean ± SEM, n = 4.

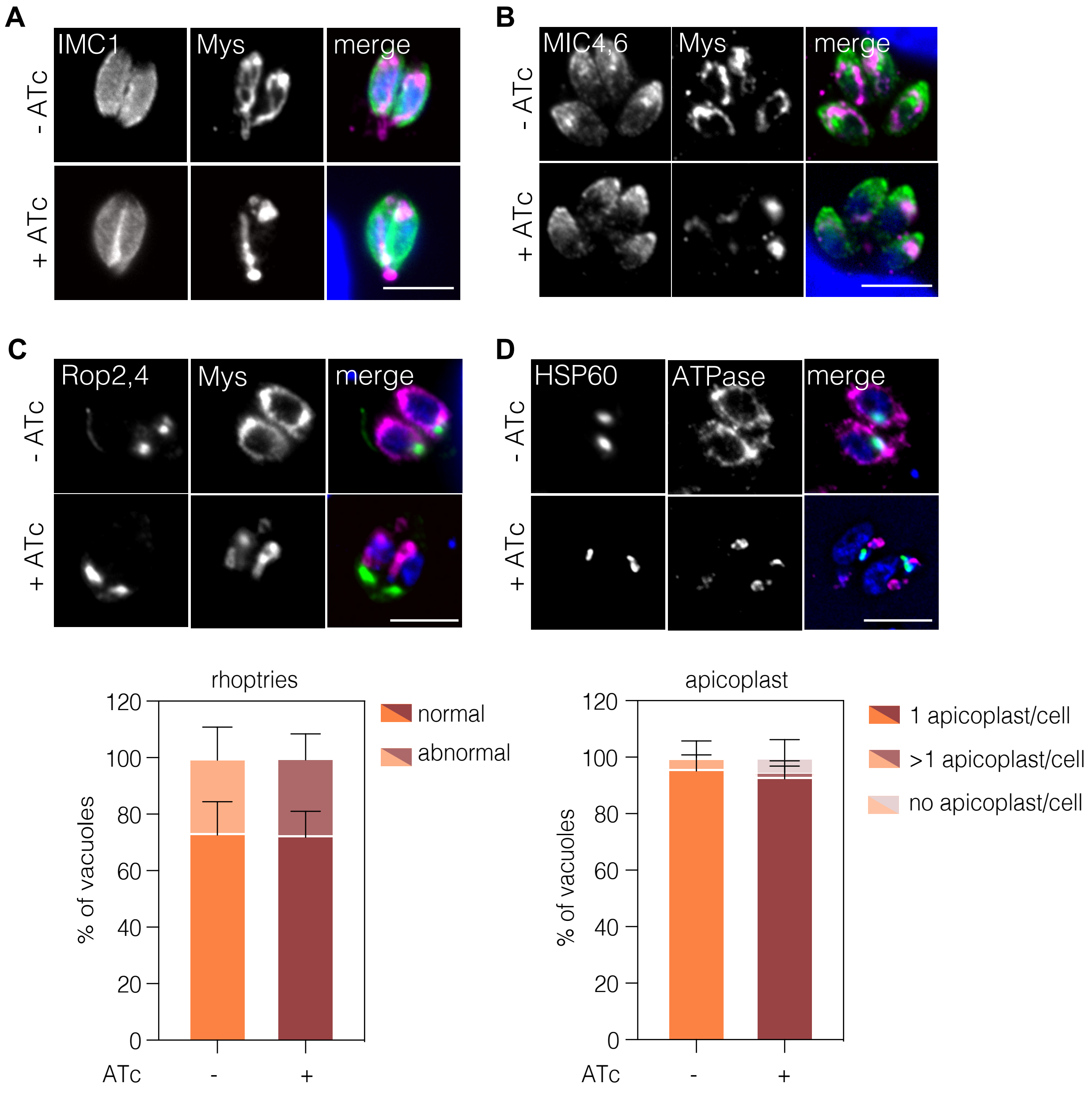

Fig. S5. Depletion of VDAC does not affect other organelles besides mitochondrion and ER
iVDAC parasites ATc treated for 48 h were fixed and stained using markers for various organelles. No difference in parasite structure (from at least 260 parasites), as visualized by anti-IMC1 **(A)** or micronemes (from at least 174 parasites), visualized using anti-MIC4, 5 **(B)** could be seen upon VDAC depletion. Imaging and quantification of the rhoptries **(C)** using anti-ROP2, 4 and the number of apicoplasts **(D)** using anti-HSP60. No significant differences could be seen in either organelle. Results mean of three independent experiments, + SD. Scale bar 5 µm.

Journal of Cell Science • Supplementary information

Table S1. List of primers used in this study

Primer number	Description	
P1	NsiI_vdac_R	cccatgcataggcacagatctcgagcttc
P2	PacI_vdac_R	cccttaattaattaggcacagatctcgagcttc
P3	VDAC_gR_F	aagttTGTACTCAACTCCGACAAAAg
P4	VDAC_gR_R	aaaacTTTTGTCGGAGTTGAGTACAa
P5	VDAC_PR_F	TCCCTCTCTCCGGGAATCTTAGCCGTTTCTGTGTACTCAACTCCGACAAA aagcttcgccaggctgtaaatcc
P6	VDAC_PR_R	CAATTTTCCGTACCCGCACAGCTTTTGTTCAAGTCCTTGAAGAGCACCAT ggttgaagacagacgaaagcagttg
P7	VDAC_test_ F	CCCTTTTTTTTCCGTTTGCGGTGTTAC
P8	DHFR_R	CACGGTTATCAAACCCGAG
P9	VDAC_test_R	CACAGAAACAAGTTTTGGGCTTTAAACACG
P10	T7S4_F	CGGTTCGCTTGAAGAAGG
P11	qVDAC_F	TGAGAAGGCATGGGACTTG
P12	qVDAC_R	CGCATATTTGACGTCGATG

Table S2. List of antibodies used in this study

**Antibody**	**Species**	**Dilution (IFA unless indicated)**	**Source**	**Reference**
CPN60 (HSP60)	Rabbit	1:1000	Boris Striepen (University of Pennsylvania)	(Agrawal et al., 2009)
IMC1	Rabbit	1:2000	Gary Ward (University of Vermont)	(Wichroski et al., 2002)
Mys (TgME49_215430)	Rabbit	1:1000		(MacRae et al., 2012)
Myc	Mouse	1:1000	Cell Signaling	
Myc	Rabbit	1:1000	ThermoFisher	
GAP45	Rabbit	1:10000	Dominique Soldati (University of Geneva)	(Plattner et al., 2008)
Sag1	Mouse	1:1000	Sebastian Lourido (Whitehead Institute)	(Burg et al., 1988)
ROP2,4	Mouse	1:500		(Sadak et al., 1988)
F1-ATPase	Mouse	1:3000	Peter Bradley (University of California, Los Angeles)	(Beck et al., 2014)
MIC4	Rabbit	1:5000		(Reiss et al., 2001)
Actin	Mouse	1:100	Markus Meissner (Ludwig-Maximilians University of Munich)	(Angrisano et al., 2012)

Table S3. Accession numbers of identified proteins.

Click here to download Table S3

Journal of Cell Science • Supplementary information
