# Supplementary material for: Depletion of a Toxoplasma porin leads to defects in mitochondrial morphology and contacts with the endoplasmic reticulum
Source: J Cell Sci. 2021 Oct 20;134(20):jcs255299. doi: 10.1242/jcs.255299 (PMC8572010; doi:10.1242/jcs.255299)
Supplement: Reviewer comments [file joces-134-255299_review_history.pdf]

# Depletion of a *Toxoplasma* porin leads to defects in mitochondrial morphology and contacts with the endoplasmic reticulum.

Natalia Mallo, Jana Ovciarikova, Erica S. Martins Duarte, Stephan C. Baehr, Marco Biddau, Mary-Louise Wilde, Alessandro D. Uboldi, Leandro Lemgruber, Christopher J. Tonkin, Jeremy G. Wideman, Clare R. Harding and Lilach Sheiner
DOI: 10.1242/jcs.255299

**Editor**: Jennifer Lippincott-Schwartz

**Review timeline**

| | |
|---|---|
| Original submission: | 7 October 2020 |
| Editorial decision: | 29 December 2020 |
| First revision received: | 19 May 2021 |
| Editorial decision: | 12 July 2021 |
| Second revision received: | 2 September 2021 |
| Accepted: | 6 September 2021 |

---

**Original submission**

First decision letter

MS ID#: JOCES/2020/255299

MS TITLE: Depletion of voltage-dependent anion channel (VDAC) of Toxoplasma gondii affects multiple mitochondrial functions, but not calcium signalling.

AUTHORS: Natalia Mallo, Erica S dos Santos Martins Duarte, Stephan C Baehr, Marco Biddau, Jana Ovciarikova, Mary-Louise Wilde, Alessandro D Uboldi, Leandro Lemgruber, Christopher J Tonkin, Jeremy G Wideman, Clare R Harding, and Lilach Sheiner
ARTICLE TYPE: Research Article

We have now reached a decision on the above manuscript.

To see the reviewers' reports and a copy of this decision letter, please go to: https://submit-jcs.biologists.org and click on the 'Manuscripts with Decisions' queue in the Author Area. (Corresponding author only has access to reviews.)

As you will see, the reviewers raise a number of substantial criticisms that prevent me from accepting the paper at this stage. They suggest, however, that a revised version might prove acceptable, if you can address their concerns. If you think that you can deal satisfactorily with the criticisms on revision, I would be pleased to see a revised manuscript. We would then return it to the reviewers. Please address all of the concerns raised by reviewer #2. The functional studies of VDAC recommended by reviewer #1 are not necessary, although your should look at whether there are any changes to mitochondrial function during VDAC depletion.

*We are aware that you may be experiencing disruption to the normal running of your lab that makes experimental revisions challenging. If it would be helpful, we encourage you to contact us to discuss your revision in greater detail. Please send us a point-by-point response indicating where you are able to address concerns raised (either experimentally or by changes to the text) and where you will not be able to do so within the normal timeframe of a revision. We will then*

© 2021. Published by The Company of Biologists under the terms of the Creative Commons Attribution License (https://creativecommons.org/licenses/by/4.0/).

*provide further guidance. Please also note that we are happy to extend revision timeframes as necessary.*

Please ensure that you clearly highlight all changes made in the revised manuscript. Please avoid using 'Tracked changes' in Word files as these are lost in PDF conversion.

I should be grateful if you would also provide a point-by-point response detailing how you have dealt with the points raised by the reviewers in the 'Response to Reviewers' box. Please attend to all of the reviewers' comments. If you do not agree with any of their criticisms or suggestions please explain clearly why this is so.

Reviewer 1

*Advance summary and potential significance to field*

In this work the authors characterized a Toxoplasma gondii gene (TgME49_263300) annotated as porin and suggested to function in anion transport. They named the gene product VDAC for voltage dependent anion channel. The authors localized the protein by transiently expressing an exogenous copy of the gene cloned into a Toxoplasma expression vector with a strong promotor and an N-terminal c-Myc tag. There was localization to sites that aligned with a mitochondrial outer membrane marker by IFA. They created conditional knockdown lines in which the expression of the gene is regulated by the addition of anhydrotetracycline (ATc) to the cultures. They show controls for integration of the promoter by PCR and downregulation of the expression of the gene by qPCR. Downregulation of VDAC caused slow growth as measured with fluorescent parasites. Additionally, downregulation of VDAC caused disruption of mitochondrial morphology as observed by IFA and also TEM. There was decrease in mitochondrial surface area and mitochondrial volume. Measurements of mitochondrial membrane potential with JC-1 found no significant differences. They did not observe a significant change in the ATP and ADP levels but the ATP/ADP ratio calculations showed a small decrease. They also evaluated global metabolic changes and they labeled parasites with 13C glucose and metabolite results showed increase of carbamoyl aspartate dihydroorotate and orotate when VDAC was downregulated. No changes in pyrimidine levels likely due to salvage. No changes in citrate, alpha-ketoglutarate, succinate, fumarate or malate. VDAC downregulation apparently affected the maturation of the mitochondrial targeted marker HSP60L-DHFR-Myc. Authors analyzed TEM images of controls and iVDAC downregulated lines and found a decrease in membrane contact sites (MCS) between the mitochondrion and the IMC and mitochondrion-ER. Additionally, they found changes in the localization, distribution and volume of the ER. They next measured cytosolic calcium with genetic indicators and found no difference in the responses to ionomycin or BIPPO. The work shows some interesting findings about the role of the gene product of the TgME49_263300 gene In Toxoplasma. However, the work is incomplete and without a clear conceptual advance, considering that no functional studies of the VDAC protein are presented.

*Comments for the author*

In animal cells VDAC is a very permissive channel through which many metabolites and ions enter and exit the mitochondria. It has also been involved in linking the ER to the mitochondria. Therefore, it is expected that downregulation of its expression results in mitochondrial damage as well as decrease in MCS, as reported here. However, it does not have a role in $Ca^{2+}$ signalling, and defects in mitochondrial membrane potential (if present) could be the consequence of the mitochondrial damage observed.

In general, this work is descriptive and some controls are needed. Mutant parasites show a growth defect but the work is missing the mechanistic reason why depletion of VDAC affects parasite growth. There are no controls for protein expression showing downregulation of VDAC with ATc. Western blot and/or IFA analyses showing the lack of expression of the protein need to be shown especially because some of the phenotypes investigated are not very clear. Reduction of expression (with qPCR) was observed at 24 h +ATc, without further changes at 48 or 72 h. Many phenotypic analyses were done at 48 hours or later and one wonders if these are related to the specific function of VDAC or just changes due to feeble cells.

© 2021. Published by The Company of Biologists under the terms of the Creative Commons Attribution License (https://creativecommons.org/licenses/by/4.0/).

Downregulation of VDAC is not expected to affect the mitochondrial membrane potential. However, JC-1 is not an ideal method for measurements of membrane potential as supported by the mitochondrial literature (PMID: 28711444).
Downregulation of VDAC is not expected to affect cytosolic calcium or calcium signaling. Considering the role of VDAC in mammalian cells it would only play a role in buffering of cytosolic calcium if a MCU were present, which apparently is not the case in T. gondii. This implies that the cytosolic calcium measurements are not that relevant. Most appropriate would be to measure mitochondrial ions/metabolite uptake which may at least suggest a biochemical function for VDAC. There are no functional studies of VDAC, which is unfortunate because it would have been important to show/suggest its molecular function.

Reviewer 2

*Advance summary and potential significance to field*

This manuscript by Mallo et al. presents the initial characterization at the cellular and metabolic levels of voltage-dependent anion channel (VDAC) in Toxoplasma. Based on homology, VDAC is predicted to be a channel in the outer membrane of the mitochondria. The authors show that depleting VDAC using regulatable knockdown leads to decreased parasite replication, extended mitochondria, and ER morphology defects.
VDAC-depleted parasites present mild defects in mitochondrial metabolism but no change in ER-derived cytosolic calcium levels. Based on changes in mitochondrial morphology and examining association by electron microscopy, the authors propose that Toxoplasma VDAC mediates membrane contact sites between the parasite mitochondria and ER.

The major advance made in this paper is showing that, similar to higher eukaryotes, Toxoplasma VDAC mediates membrane contact sites between the mitochondria and ER. The results presented in this manuscript added important aspects of mitochondrial proteins in maintain mitochondria morphology, but some data and conclusions are premature and speculative. Unfortunately, the role of VDAC-mediate membrane contact sites during parasite infection are not completely described, in particular the lack of a mechanism to explain the replication defect.

*Comments for the author*

Major comments
1. The proposed function of VDAC requires that it is found in the mitochondrial outer membrane. While the immunofluorescence microscopy demonstrates that VDAC co-localizes with the mitochondrial outer membrane protein marker TgMys, this approach does not provide the necessary resolution to differentiate the outer and inner mitochondrial membranes. It needs to be established that VDAC is indeed the outer membrane using a technique such as digitonin permeabilization (Jacobs et al 2020).

2. Given that VDAC depletion results in only mild defects on mitochondrial function and calcium levels, the severe replication defect may primarily be related to the change in mitochondrial morphology. However, the current study does not establish why maintaining mitochondria-ER membrane contact sites is essential for replication. A more thorough investigation of parasite replication in VDAC-depleted parasites, including a doubling assay and examining mitochondrial segregation during endodyogeny may help establish why VDAC is essential.

3. Correct confirmation of 5’ and 3’ integration of the iVDAC parasites is crucial. In Fig. 1, it is not clear where the primers are located in Fig 1B, the expected sizes of PCR products, and why multiple bands are present in Fig 1C. More specific primers should be used to confirm generation of the iVDAC parasites. The PCR for 3’ integration shows multiple undefined bands, and it appears that the gel was cut and processed to put the iVDAC and parental lanes next to each other. If this is the case, please add a line between lanes to show that the gel was cut.

4. The ATc system was used to control mRNA levels of VDAC, however, VDAC protein levels were not evaluated. Thus, it is not clear the level and rate at which VDAC protein levels were depleted in presence of ATc. Including an endogenous tag (e.g., myc as in Fig. 1A) during promoter

© 2021. Published by The Company of Biologists under the terms of the Creative Commons Attribution License (https://creativecommons.org/licenses/by/4.0/). 

replacement would enable analysis of VDAC protein levels. This is especially important as it appears that basal VDAC mRNA levels are higher in the iVDAC parasites, compared to parental (see next comment).

5. In Fig. 1D, it appears that iVDAC parasites have more basal VDAC mRNA (0 hr ATc) compared to WT. Is there any statistical significance in VDAC mRNA between parental and iVDAC at 0h? Along the same lines, is there a significant difference in the percentange of parasites with 5. connected mitochondria at 0h between parental and iVDAC parasites. This difference might be correlated to different VDAC expression levels in iVDAC and parental parasites.

Minor comments.
Line 47: Missing reference.
Line 102-103: The conservation of VDAC in Apicomplexans is overstated. While TgVDAC is highly conserved with H. hammondi and N. caninum, there is only 29% homology with P. falciparum.
Line 115: Add the CRISPR score value (-3.67).
Line 116: Include (iVDAC) after “conditional knockdown line by replacing the VDAC”.
Lines 123: Please change word “fitness” to replication.
Fig. 1D and E: What is the expression level at 5, 6, and 7 days post infection? iVDAC+ATc parasites start to show difference in replication after 5 days of incubation with ATc, however the RT-qPCR was only performed out to 3 days.
Fig 3B: The increase in abnormal morphologies would be more obvious if “normal” and “abnormal” were different colors.
Fig 4A. The experiment was done once, with 94 parasites analyzed. It is not clear whether the counts were blinded or not. Detail if the TEM photos were blinded before counting to reduce bias.
Fig. 4B: The authors could provide information whether the counting was blinded or not.
Fig.4A: It will help visualization if the authors draw a color line on the mitochondria and ER membranes.
Fig.5: The title states that VDAC depletion affects Ca2+ induced motility, however this contradicts lines 290-
291, and Fig 5C shows no differences between - and +ATc.

---

**First revision**

Author response to reviewers' comments

**Dear Jennifer,**

**We like to thank you and the reviewers for taking the time to consider our manuscript for publication in the Journal of Cell Science, and for making suggestions that have improved the manuscript. We further thank you for the extension granted to allow us to tackle nearly all suggestions with new experiments despite the restrictions imposed by the pandemic. We had considered each of the comments carefully and where possible included new experiments. Please see below our point by point respond to the reviewers comments, where the comments are in black text and our responds are in green and bold.**

Reviewer 1 Advance Summary and Potential Significance to Field:

In this work the authors characterized a Toxoplasma gondii gene (TgME49_263300) annotated as porin and suggested to function in anion transport. They named the gene product VDAC for voltage dependent anion channel. The authors localized the protein by transiently expressing an exogenous copy of the gene cloned into a Toxoplasma expression vector with a strong promotor and an N-terminal c-Myc tag. There was localization to sites that aligned with a mitochondrial outer membrane marker by IFA. They created conditional knockdown lines in which the expression of the gene is regulated by the addition of anhydrotetracycline (ATc) to the cultures. They show controls for integration of the promoter by PCR and downregulation of the expression of the gene by qPCR. Downregulation of VDAC caused slow growth as measured with fluorescent parasites. Additionally,

© 2021. Published by The Company of Biologists under the terms of the Creative Commons Attribution License (https://creativecommons.org/licenses/by/4.0/). 

downregulation of VDAC caused disruption of mitochondrial morphology as observed by IFA and also TEM. There was decrease in mitochondrial surface area and mitochondrial volume. Measurements of mitochondrial membrane potential with JC-1 found no significant differences. They did not observe a significant change in the ATP and ADP levels but the ATP/ADP ratio calculations showed a small decrease. They also evaluated global metabolic changes and they labeled parasites with 13C glucose and metabolite results showed increase of carbamoyl aspartate, dihydroorotate and orotate when VDAC was downregulated. No changes in pyrimidine levels likely due to salvage. No changes in citrate, alpha-ketoglutarate, succinate, fumarate or malate. VDAC downregulation apparently affected the maturation of the mitochondrial targeted marker HSP60L-DHFR-Myc. Authors analyzed TEM images of controls and iVDAC downregulated lines and found a decrease in membrane contact sites (MCS) between the mitochondrion and the IMC and mitochondrion-ER. Additionally, they found changes in the localization, distribution and volume of the ER. They next measured cytosolic calcium with genetic indicators and found no difference in the responses to ionomycin or BIPPO. The work shows some interesting findings about the role of the gene product of the TgME49_263300 gene In Toxoplasma. However, the work is incomplete and without a clear conceptual advance, considering that no functional studies of the VDAC protein are presented.

**In our view, a major conceptual advance is in providing evidence for the existence of ER-mitochondria membrane contact sites in Toxoplasma, and support for VDAC's involvement in mediating them. These findings are important and timely as membrane contact sites are largely understudied in these organisms despite growing understanding of their composition and function in common model organisms. We were happy that reviewer 2 agreed with us about this point.**

Reviewer 1 Comments for the Author:

In animal cells VDAC is a very permissive channel through which many metabolites and ions enter and exit the mitochondria. It has also been involved in linking the ER to the mitochondria. Therefore, it is expected that downregulation of its expression results in mitochondrial damage as well as decrease in MCS, as reported here.

**Fundamental differences between opisthokont (where VDAC has been studied so far) and apicomplexan cell biology are constantly uncovered. We believe no finding of similar observation between these lineages is expected, and that new studies in apicomplexan are essential for full understanding of cell biology.**
**Moreover, in animal cell, VDAC's involvement in ER-mitochondrial MCS occurs through interaction with partners that are missing in Apicomplexa ((Bick et al., 2012) and our Figures 2E and S3). Therefore, its role in ER mitochondria MCS in these parasites was an open question, which our study addressed.**

However, it does not have a role in Ca2+ signalling, and defects in mitochondrial membrane potential (if present) could be the consequence of the mitochondrial damage observed.

**We removed the reference to $Ca^{2+}$ signalling from the title, and restructured the text to provide a clearer context to the reasons for which we assessed $Ca^{2+}$ signalling (line 168-170) and membrane potential (lines 418-422) under VDAC depletion.**

In general, this work is descriptive and some controls are needed. Mutant parasites show a growth defect but the work is missing the mechanistic reason why depletion of VDAC affects parasite growth.

**We now provide new experiments that break down the temporal phenotypes and identify that mitochondrial morphology defect leads to ER collapse and results in parasite replication defect leading to the overall growth defect (Figure 1J, new time points added to Figure 2B, and figure 5H).**

There are no controls for protein expression showing downregulation of VDAC with ATc. Western blot and/or IFA analyses showing the lack of expression of the protein need to be shown especially because some of the phenotypes investigated are not very clear.

© 2021. Published by The Company of Biologists under the terms of the Creative Commons Attribution License (https://creativecommons.org/licenses/by/4.0/). 

**The revised Figure 1 now includes new panels with Western blot of endogenously tagged VDAC during depletion and its quantification (Figure 1D-F).**

Reduction of expression (with qPCR) was observed at 24h +ATc, without further changes at 48 or 72 h. Many phenotypic analyses were done at 48 hours or later and one wonders if these are related to the specific function of VDAC or just changes due to feeble cells.

**We measured the majority of phenotypes at 48 h to ensure that remaining VDAC protein was reduced (Figure 1F). Although VDAC is clearly essential, we believe that the parasite's normal metabolite levels of the TCA cycle (Figure 4E), the lack of change in the mitochondrial membrane potential (Figure 4A) and ability to move and invade normally (Figure 2A,B) demonstrate that these parasites are not generally 'feeble' but that the responses that we do see are specific to the depletion of VDAC.**

Downregulation of VDAC is not expected to affect the mitochondrial membrane potential. However, JC-1 is not an ideal method for measurements of membrane potential as supported by the mitochondrial literature (PMID: 28711444).

**Membrane potential was used to indirectly assess any defect in mitochondrial translation, in light of evidence in other organisms implicating VDAC in tRNA import. This is explained in lines 418-422. JC-1 is standard in the *Toxoplasma* field for mitochondrial potential measurements (PMIDs: 20036630; 33402698; 33651838). For example, in our recent PLOS pathogens paper we used this method to detect a defect in mitochondrial membrane potential under mETC complex depletion.**
**For clarity, we have added the caveat raised in the cited 28711444 in our discussion of the JC-1 observations (line 423-425).**

Downregulation of VDAC is not expected to affect cytosolic calcium or calcium signaling. Considering the role of VDAC in mammalian cells it would only play a role in buffering of cytosolic calcium if a MCU were present, which apparently is not the case in T. gondii. This implies that the cytosolic calcium measurements are not that relevant.

**While through phylogenetic approaches we do not find a canonical MCU (Figures 2E and S3), this does not mean that there is not a possible calcium transporter performing this role. Since the role of VDAC is associated with calcium in a number of species, we decided it was worthwhile to examine calcium even in the absence of a predicted MCU.**

Most appropriate would be to measure mitochondrial ions/metabolite uptake which may at least suggest a biochemical function for VDAC. There are no functional studies of VDAC, which is unfortunate because it would have been important to show/suggest its molecular function.

**We agree that biochemical functional studies will be required to understand the basis of substrate specificity between the *Toxoplasma* VDAC and VDACs from other species, however we feel that this is beyond the scope of this study, which provides extensive cellular and molecular biology characterization.**

Reviewer 2 Advance Summary and Potential Significance to Field:

This manuscript by Mallo et al. presents the initial characterization at the cellular and metabolic levels of voltage-dependent anion channel (VDAC) in Toxoplasma. Based on homology, VDAC is predicted to be a channel in the outer membrane of the mitochondria. The authors show that depleting VDAC using regulatable knockdown leads to decreased parasite replication, extended mitochondria, and ER morphology defects. VDAC-depleted parasites present mild defects in mitochondrial metabolism but no change in ER- derived cytosolic calcium levels. Based on changes in mitochondrial morphology and examining association by electron microscopy, the authors propose that Toxoplasma VDAC mediates membrane contact sites between the parasite mitochondria and ER.

The major advance made in this paper is showing that, similar to higher eukaryotes, Toxoplasma VDAC mediates membrane contact sites between the mitochondria and ER. The results presented

© 2021. Published by The Company of Biologists under the terms of the Creative Commons Attribution License (https://creativecommons.org/licenses/by/4.0/).

in this manuscript added important aspects of mitochondrial proteins in maintain mitochondria morphology, but some data and conclusions are premature and speculative. Unfortunately, the role of VDAC-mediate membrane contact sites during parasite infection are not completely described, in particular the lack of a mechanism to explain the replication defect.

**We thank the reviewer for highlighting the importance of our work. In the revised manuscript we had addressed the mechanistic question experimentally (details explained below).**

Major comments

1. The proposed function of VDAC requires that it is found in the mitochondrial outer membrane. While the immunofluorescence microscopy demonstrates that VDAC co- localizes with the mitochondrial outer membrane protein marker TgMys, this approach does not provide the necessary resolution to differentiate the outer and inner mitochondrial membranes. It needs to be established that VDAC is indeed the outer membrane using a technique such as digitonin permeabilization (Jacobs et al 2020).

**We now provide evidence for VDAC's outer membrane localization using a DuoLink proximity ligation amplification system, which amplified a signal of endogenously tagged VDAC's proximity to the known outer membrane proteins TgMys and TOM40. As a control we show that the matrix mitoribosomal protein TgmS35 does not produce signal of proximity to TgMys or TOM (Figure 1H/I and Figure S2B).**

2. Given that VDAC depletion results in only mild defects on mitochondrial function and calcium levels, the severe replication defect may primarily be related to the change in mitochondrial morphology. However, the current study does not establish why maintaining mitochondria-ER membrane contact sites is essential for replication. A more thorough investigation of parasite replication in VDAC-depleted parasites, including a doubling assay and examining mitochondrial segregation during endodyogeny may help establish why VDAC is essential.

**We now provide detailed temporal analysis of doubling time (Figure 1J). For further investigation of mitochondria during parasite replication, we attempted live imaging, which we found inconclusive (attached figures here for the reviewer to evaluate). Instead, we provided detailed temporal investigation of mitochondrial and ER morphology changes upon VDAC depletion defect (Figure 5H). The new replication and morphology analysis data leads to a proposed mechanism which we discuss in line 459-464.**

3. Correct confirmation of 5' and 3' integration of the iVDAC parasites is crucial. In Fig. 1, it is not clear where the primers are located in Fig 1B, the expected sizes of PCR products, and why multiple bands are present in Fig 1C. More specific primers should be used to confirm generation of the iVDAC parasites. The PCR for 3' integration shows multiple undefined bands, and it appears that the gel was cut and processed to put the iVDAC and parental lanes next to each other. If this is the case, please add a line between lanes to show that the gel was cut.

**We apologies, and we have replaced this gel with a new version, which was not cut and shows correct integration, we removed the non-specific bands by increasing the annealing temperature (Figure 1B), and the expected size of PCR products is now indicated on Figure 1A.**

4. The ATc system was used to control mRNA levels of VDAC, however, VDAC protein levels were not evaluated. Thus, it is not clear the level and rate at which VDAC protein levels were depleted in presence of ATc. Including an endogenous tag (e.g., myc as in Fig. 1A) during promoter replacement would enable analysis of VDAC protein levels. This is especially important as it appears that basal VDAC mRNA levels are higher in the iVDAC parasites, compared to parental (see next comment).

**We tagged VDAC endogenously in the promoter replacement line and Figure 1E,F now shows down regulation of protein levels via western.**

5. In Fig. 1D, it appears that iVDAC parasites have more basal VDAC mRNA (0 hr ATc) compared to WT. Is there any statistical significance in VDAC mRNA between parental and iVDAC at 0h? Along

© 2021. Published by The Company of Biologists under the terms of the Creative Commons Attribution License (https://creativecommons.org/licenses/by/4.0/). 

the same lines, is there a significant difference in the percentange of parasites with connected mitochondria at 0h between parental and iVDAC parasites. This difference might be correlated to different VDAC expression levels in iVDAC and parental parasites.

**We cannot compare VDAC expression of parental and iVDAC no ATc as the series of RT-qPCR time points for each line was done separately. To prevent confusion we separated the two graphs in Figure 1C. As for the connected mitochondria, the difference is not significant.**

Minor comments.

Line 47: Missing reference.

**Thank you for pointing this out. This is now line 49 and the references are added.**

Line 102-103: The conservation of VDAC in Apicomplexans is overstated. While TgVDAC is highly conserved with H. hammondi and N. caninum, there is only 29% homology with P. falciparum.

**We apologies for creating a misunderstanding in how we wrote about homology (similarity due to common ancestry) versus sequence identity/similarity. We have changed the table heading in Figure S1B and changed the sentence to read "VDAC is conserved between Apicomplexa, and while sequence identity between orthologues can drop below 30% (Figure S1B), VDAC is nearly ubiquitously conserved across all eukaryotes (Wideman et al. 2013)."**

Line 115: Add the CRISPR score value (-3.67).

**This has been done (line 109 now), thank you**

Line 116: Include (iVDAC) after "conditional knockdown line by replacing the VDAC".

**We have done this, the line (128) now reads: we constructed a conditional knockdown line (named iVDAC for inducible VDAC)**

Lines 123: Please change word "fitness" to replication.

**This has been done.**

Fig. 1D and E: What is the expression level at 5, 6, and 7 days post infection? iVDAC+ATc parasites start to show difference in replication after 5 days of incubation with ATc, however the RT-qPCR was only performed out to 3 days.

**We now present RT-qPCR on day 5 showing continued low level of expression (Figure 1C), similar to days 1-3. Additionally, in our new analysis of replication, defect is observed already at 48 h (Figure 1J).**

Fig 3B: The increase in abnormal morphologies would be more obvious if "normal" and "abnormal" were different colors.

**Thank you for this suggestion, we have changed the 'abnormal' morphologies to shades of teal, which should hopefully help with interpretation. These data is now in Figure 3A,B.**

Fig 4A. The experiment was done once, with 94 parasites analyzed. It is not clear whether the counts were blinded or not. Detail if the TEM photos were blinded before counting to reduce bias. Fig. 4B: The authors could provide information whether the counting was blinded or not.

**The counting was done unblinded in both cases.**

Fig.4A: It will help visualization if the authors draw a color line on the mitochondria and ER membranes.

© 2021. Published by The Company of Biologists under the terms of the Creative Commons Attribution License (https://creativecommons.org/licenses/by/4.0/).

**We have added a panel with the relevant membranes in colour, thank you. These data is now in Figure 5A.**

Fig.5: The title states that VDAC depletion affects Ca2+ induced motility, however this contradicts lines 290- 291, and Fig 5C shows no differences between – and +ATc.

**We agree. We have modified the title, and re-structured the manuscript such that the defects and unchanged phenotypes are describes in a manner that clarifies the sequence of events.**

Figure 1 – live imaging of iVDAC at 24 h after addition of ATC (mitochondria in green)

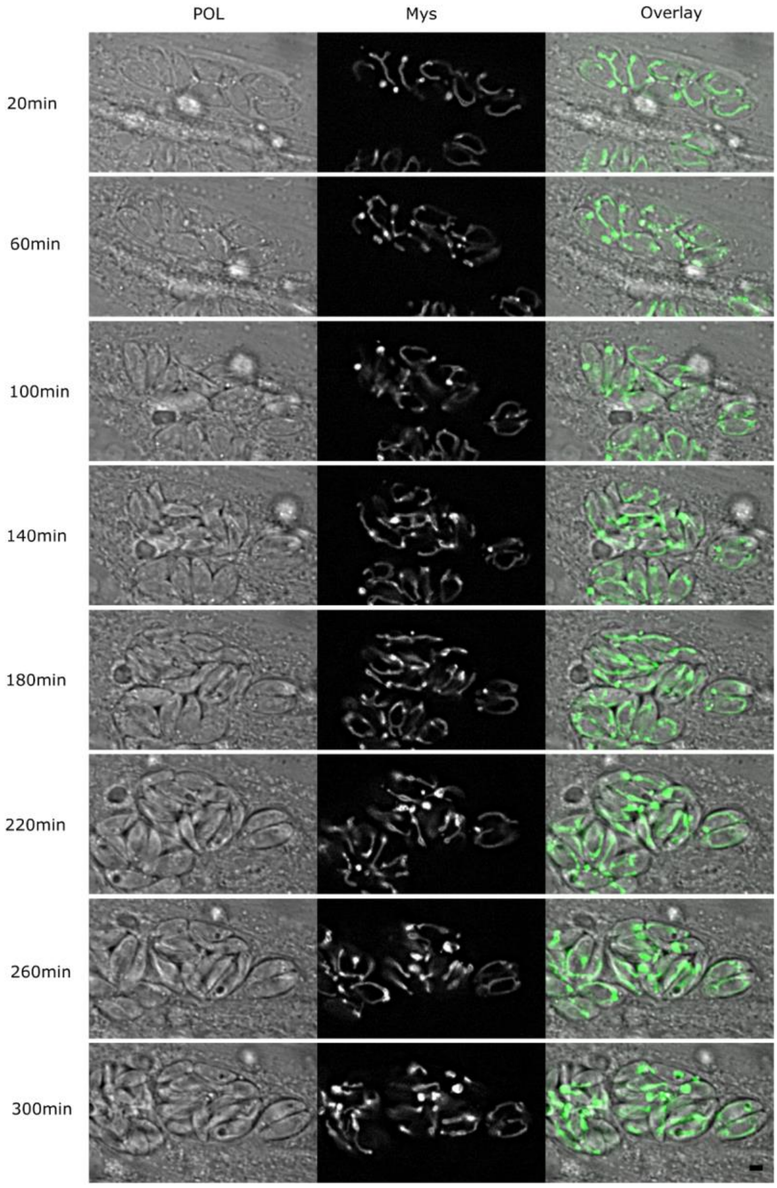


© 2021. Published by The Company of Biologists under the terms of the Creative Commons Attribution License (https://creativecommons.org/licenses/by/4.0/).

Figure 2 – live imaging of iVDAC at 48 h after addition of ATC (mitochondria in green)

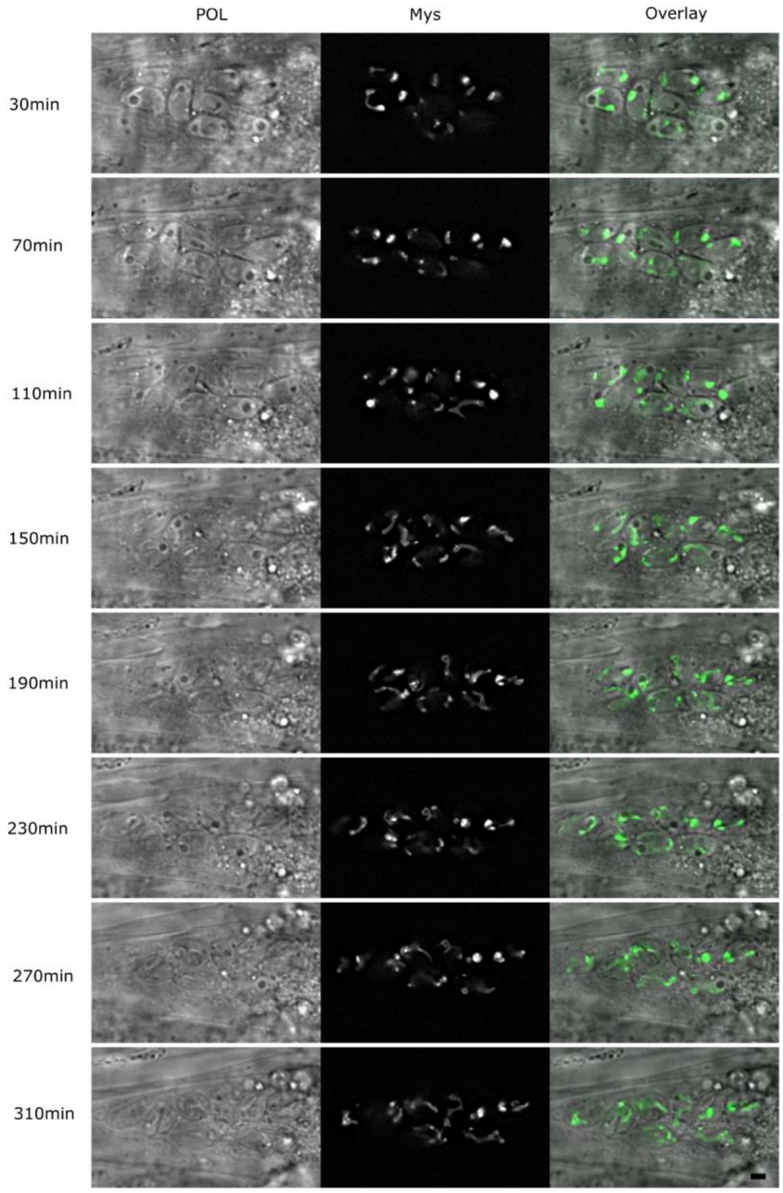


---

Second decision letter

MS ID#: JOCES/2020/255299

MS TITLE: Depletion of voltage-dependent anion channel (VDAC) of Toxoplasma gondii affects multiple mitochondrial functions, but not calcium signalling.

AUTHORS: Natalia Mallo, Jana Ovciarikova, Erica S dos Santos Martins Duarte, Stephan C Baehr, Marco Biddau, Mary-Louise Wilde, Alessandro D Uboldi, Leandro Lemgruber, Christopher J Tonkin, Jeremy G Wideman, Clare R Harding, and Lilach Sheiner
ARTICLE TYPE: Research Article

We have now reached a decision on the above manuscript.

© 2021. Published by The Company of Biologists under the terms of the Creative Commons Attribution License (https://creativecommons.org/licenses/by/4.0/).

To see the reviewers' reports and a copy of this decision letter, please go to: https://submit-jcs.biologists.org and click on the 'Manuscripts with Decisions' queue in the Author Area. (Corresponding author only has access to reviews.)

As you will see, the reviewers gave favourable reports but raised some critical points that will require amendments to your manuscript. I hope that you will be able to carry these out because I would like to be able to accept your paper, depending on further comments from reviewers.

*We are aware that you may be experiencing disruption to the normal running of your lab that makes experimental revisions challenging. If it would be helpful, we encourage you to contact us to discuss your revision in greater detail. Please send us a point-by-point response indicating where you are able to address concerns raised (either experimentally or by changes to the text) and where you will not be able to do so within the normal timeframe of a revision. We will then provide further guidance. Please also note that we are happy to extend revision timeframes as necessary.*

Please ensure that you clearly highlight all changes made in the revised manuscript. Please avoid using 'Tracked changes' in Word files as these are lost in PDF conversion.

I should be grateful if you would also provide a point-by-point response detailing how you have dealt with the points raised by the reviewers in the 'Response to Reviewers' box. Please attend to all of the reviewers' comments. If you do not agree with any of their criticisms or suggestions please explain clearly why this is so.

Reviewer 1

*Advance summary and potential significance to field*

The authors characterized the Voltage Dependent Anion Channel orthologue of Toxoplasma gondii (TgME49_263300) which is annotated as porin and suggested to function in anion transport.

In this resubmission the authors present new controls for the regulation of the expression of TgVDAC by showing western blots of the endogenously tagged gene.
The authors localised the protein by HA C-terminus tagging and also by transiently expressing an exogenous copy of the gene cloned into a Toxoplasma expression vector with a strong promoter and an N-terminal c-Myc tag. They also use DuoLink proximity ligation amplification (PLA) system to show localisation to the outer membrane of the mitochondria. To determine if VDAC was found on the outer mitochondrial membrane, they used the endogenously tagged VDAC and antibodies against two proteins known to be in the outer mitochondrial membrane Mys and TOM40. They saw specific amplification of the signal with the outer membrane markers. Authors analysed TEM images of controls and iVDAC downregulated lines and found a decrease in membrane contact sites (MCS) between the mitochondrion and the IMC and mitochondrion-ER. Additionally, they found changes in the localisation, distribution and volume of the ER.

*Comments for the author*

1. This is an improved version of the previous manuscript. The authors state that their major contribution is the potential role of VDAC in the establishment of MCS between the mitochondrion and the ER of Toxoplasma.
Considering this, I think that it should be appropriate to cite previous work about the presence of MCS between the ER and other organelles like the apicoplast: Tomova C, et al. 2009. Membrane contact sites between apicoplast and ER in Toxoplasma gondii revealed by electron tomography. Traffic 10, 1471-1480.

2. Second, and more importantly, the data presented in Figures 5A and B becomes an essential part of the paper. I think that the data showing MCS formation should include statistical analysis of the difference between the control and the downregulated mutant. This will present a more convincing story and will strengthen the conclusions of the manuscript. Also, it would be

© 2021. Published by The Company of Biologists under the terms of the Creative Commons Attribution License (https://creativecommons.org/licenses/by/4.0/).

important to include an analysis of the % of mitochondria with membrane apposition in the +ATc mutant. There is no mention of the number of independent biological samples analysed.

3. If VDAC is involved in Ca2+ mobilisation, as considered in the paper and the references cited, this could be through MCS and Ca2+ would be allowed to flow through the outer membrane to be available for the unknown transport mechanism in the inner mitochondrial membrane. Ca2+ would be probably transferred from the ER to the mitochondria considering that the ER is the most likely organelle with the highest Ca2+. In other cells, VDAC is mostly involved in the uptake of Ca2+ playing a role in homeostasis rather than signalling as stated in the paper. Considering that Ca2+ would be transported from the ER to the mitochondria it is not clear how VDAC function would impact cytosolic Ca2+.

4. In addition, the Ca2+ graphs presented would need to be analysed. The legend does not indicate the number of experiments and if the cytosolic concentration of Ca2+ was measured. From the single graphs presented it appears that there could be a difference in the response to BIPPO which could become evident after analysing the slopes.

5. If VDAC plays a role in Ca2+ transport (most likely entry into the mitochondria) then the experiments to test would be the response of cytosolic Ca2+ to mitochondrial inhibitors like FCCP or antimycin A. If the mitochondria play a role in taking up Ca2+ or if Ca2+ is released through VDAC then it would be appropriate to test mitochondrial inhibitors.

6. Considering the role of the mitochondria in other cells as a Ca2+ buffer, experiments testing the sensitivity of the mutants to Ca2+ overload would be appropriate. This phenotype has not been tested.

7. In conclusion the Ca2+ phenotypes that would define a role for VDAC in Ca2+ homeostasis have not been tested so authors should tone down their conclusions about Ca2+ in the abstract, introduction, and discussion.

Reviewer 2

*Advance summary and potential significance to field*

The resubmission addresses the primary concerns, and provides new data which gives more mechanistic insight into mitochondrial-ER membrane contact sites in Toxoplasma.

*Comments for the author*

Figure 1F and line 129: Is it statistically significant? If not, remove the word “a significant” from line 129; and also add at line 518 the number of replicates (n=?)

---

**Second revision**

Author response to reviewers' comments

Dear Jennifer,

Once again, we thank you and the reviewers for the helpful feedback on our manuscript. We were happy to see that the reviewers agree that the manuscript is improved, and the new feedback helped us finalise it.
Below please find our point by point respond (blue/bold) to the reviewer’s new comments (black).

Reviewer 1 Advance Summary and Potential Significance to Field:
The authors characterized the Voltage Dependent Anion Channel orthologue of Toxoplasma gondii

© 2021. Published by The Company of Biologists under the terms of the Creative Commons Attribution License (https://creativecommons.org/licenses/by/4.0/).

(TgME49_263300) which is annotated as porin and suggested to function in anion transport. In this resubmission the authors present new controls for the regulation of the expression of TgVDAC by showing western blots of the endogenously tagged gene. The authors localised the protein by HA C-terminus tagging and also bytransiently expressing an exogenous copy of the gene cloned into a Toxoplasma expression vector with a strong promoter and an N-terminal c-Myc tag. They also use DuoLink proximity ligation amplification (PLA) system to show localisation to the outer membrane of the mitochondria. To determine if VDAC was found on the outer mitochondrial membrane, they used the endogenously tagged VDAC and antibodies against two proteins known to be in the outer mitochondrial membrane, Mys and TOM40. They saw specific amplification of the signal with the outer membrane markers. Authors analysed TEM images of controls and iVDAC downregulated lines and found a decrease in membrane contact sites (MCS) between the mitochondrion and the IMC and mitochondrion-ER. Additionally, they found changes in the localisation, distribution and volume of the ER.

Reviewer 1 Comments for the Author:
1. This is an improved version of the previous manuscript.

The authors state that their major contribution is the potential role of VDAC in the establishment of MCS between the mitochondrion and the ER of Toxoplasma. Considering this, I think that it should be appropriate to cite previous work about the presence of MCS between the ER and other organelles like the apicoplast: Tomova C, et al. 2009. Membrane contact sites between apicoplast and ER in Toxoplasma gondii revealed by electron tomography. Traffic 10, 1471-1480.

**This was added to the discussion (line 432-3)**

2. Second, and more importantly, the data presented in Figures 5A and B becomes an essential part of the paper. I think that the data showing MCS formation should include statistical analysis of the difference between the control and the downregulated mutant. This will present a more convincing story and will strengthen the conclusions of the manuscript. Also, it would be important to include an analysis of the % of mitochondria with membrane apposition in the +ATc mutant. There is no mention of the number of independent biological samples analysed.

**We agree with the reviewer. We thus performed additional TEM experiments to provide this data which is now included in the revised Figure 5B.**

3. If VDAC is involved in Ca2+ mobilisation, as considered in the paper and the references cited, this could be through MCS and Ca2+ would be allowed to flow through the outer membrane to be available for the unknown transport mechanism in the inner mitochondrial membrane. Ca2+ would be probably transferred from the ER to the mitochondria considering that the ER is the most likely organelle with the highest Ca2+. In other cells, VDAC is mostly involved in the uptake of Ca2+ playing a role in homeostasis rather than signalling as stated in the paper. Considering that Ca2+ would be transported from the ER to the mitochondria it is not clear how VDAC function would impact cytosolic Ca2+.

**We agree with the reviewer that the ins and outs of calcium homeostasis is not fully clear, however since it is not the focus of our paper, we avoid discussing this in length and instead we followed the reviewer's comment no. 7 - see below.**
**We also changed "signalling" to homeostasis - lines: 66, 371, 465, 479.**

4. In addition, the Ca2+ graphs presented would need to be analysed. The legend does not indicate the number of experiments and if the cytosolic concentration of Ca2+ was measured. From the single graphs presented it appears that there could be a difference in the response to BIPPO which could become evident after analysing the slopes.

**We thank the reviewer for suggesting this additional analysis, which we had performed. Our analysis of 3 repetitions indeed highlighted a difference in cytosolic calcium following BIPPO treatment upon VDAC depletion. This is now represented with new graphs in the revised Figure 2D and discussed in lines 164, and 188-191.**

© 2021. Published by The Company of Biologists under the terms of the Creative Commons Attribution License (https://creativecommons.org/licenses/by/4.0/).

5. If VDAC plays a role in Ca2+ transport (most likely entry into the mitochondria) then the experiments to test would be the response of cytosolic Ca2+ to mitochondrial inhibitors like FCCP or antimycin A. If the mitochondria play a role in taking up Ca2+ or if Ca2+ is released through VDAC then it would be appropriate to test mitochondrial inhibitors.
6. Considering the role of the mitochondria in other cells as a Ca2+ buffer, experiments testing the sensitivity of the mutants to Ca2+ overload would be appropriate. This phenotype has not been tested.
7. In conclusion the Ca2+ phenotypes that would define a role for VDAC in Ca2+ homeostasis have not been tested so authors should tone down their conclusions about Ca2+ in the abstract, introduction, and discussion.

**Since our focus is on the role of VDAC in mitochondrial-ER contact, and the effect on calcium is a small part of the results reported, we feel that the newly proposed experiments in point 5 and 6 for this second revision are out of the scope of the paper.**
**We thus followed the reviewer's suggestion in point 7 and have revised our text: We removed the point about calcium from the abstract (line 34).**
**We revise the conclusion comment in the introduction about calcium (lines 96-101) We revised the conclusion in the discussion (lines 477-480).**

Reviewer 2 Advance Summary and Potential Significance to Field:

The resubmission addresses the primary concerns, and provides new data which gives more mechanistic insight into mitochondrial-ER membrane contact sites in Toxoplasma.

Reviewer 2 Comments for the Author:

Figure 1F and line 129: Is it statistically significant? If not, remove the word “a significant” from line 129; and also add at line 518 the number of replicates (n=?)

**The difference shown is significant for both time points compared to time 0. We added the P values in the revise Figure 1. We had provided the repetition number (n=3) as proposed (line 509).**

Manuscript colour coding: yellow - first round of revisions; green: new revisions corresponding to the above.

---

Third decision letter

MS ID#: JOCES/2020/255299

MS TITLE: Depletion of voltage-dependent anion channel (VDAC) of Toxoplasma gondii affects multiple mitochondrial functions, but not calcium signalling.

AUTHORS: Natalia Mallo, Jana Ovciarikova, Erica S dos Santos Martins Duarte, Stephan C Baehr, Marco Biddau, Mary-Louise Wilde, Alessandro D Uboldi, Leandro Lemgruber, Christopher J Tonkin, Jeremy G Wideman, Clare R Harding, and Lilach Sheiner
ARTICLE TYPE: Research Article

I am happy to tell you that your manuscript has been accepted for publication in Journal of Cell Science, pending standard ethics checks.

© 2021. Published by The Company of Biologists under the terms of the Creative Commons Attribution License (https://creativecommons.org/licenses/by/4.0/).
